# On the measurement of cause of death inequality

**DOI:** 10.1093/ije/dyae016

**Published:** 2024-02-14

**Authors:** Iñaki Permanyer, Júlia Almeida Calazans

**Affiliations:** Centre for Demographic Studies, Centres de Recerca de Catalunya (CERCA), Universitat Autònoma de Barcelona, Bellaterra, Spain; ICREA, Passeig LLuís Companys 23, Barcelona, Spain; Centre for Demographic Studies, Centres de Recerca de Catalunya (CERCA), Universitat Autònoma de Barcelona, Bellaterra, Spain

**Keywords:** Cause of death, diversity, dissimilarity, mortality profile, heterogeneity, mortality inequality

## Abstract

**Background:**

Attempts at assessing heterogeneity in countries’ mortality profiles often rely on measures of cause of death (CoD) diversity. Unfortunately, such indicators fail to take into consideration the degree of (dis)similarity among pairs of causes (e.g. ‘transport injuries’ and ‘unintentional injuries’ are implicitly assumed to be as dissimilar as ‘transport injuries’ and ‘Alzheimer’s disease’)－an unrealistic and unduly restrictive assumption.

**Development:**

We extend diversity indicators proposing a broader class of heterogeneity measures that are sensitive to the similarity between the causes of death one works with. The so-called ‘CoD inequality’ measures are defined as the average expected ‘dissimilarity between any two causes of death’. A strength of the approach is that such measures are decomposable, so that users can assess the contribution of each cause to overall CoD heterogeneity levels—a useful property for the evaluation of public health policies.

**Application:**

We have applied the method to 15 low-mortality countries between 1990 and 2019, using data from the Global Burden of Disease project. CoD inequality and CoD diversity generally increase over time across countries and sex, but with some exceptions. In several cases (notably, Finland), both indicators run in opposite directions.

**Conclusions:**

CoD inequality and diversity indicators capture complementary information about the heterogeneity of mortality profiles, so they should be analysed alongside other population health metrics, such as life expectancy and lifespan inequality.

Key Messages

- Current assessments of heterogeneity in mortality profiles rely on measures of cause of death (CoD) diversity, which are insensitive to the degree of (dis)similarity that might exist among pairs of CoD.
- The proposed CoD inequality indicators measure the average dissimilarity among CoD pairs, generalize currently existing diversity measures and are decomposable by cause.
- Applications of the new methodology provide complementary information about the heterogeneity in mortality profiles, thus presenting valuable insights into the evaluation and design of public health policies.

## Introduction

In the study of contemporary health dynamics, much is known about the main causes from which individuals die.^1–7^ As the epidemiological transition unfolded, there were dramatic changes in mortality profiles around the world—with a shift from a preponderance of communicable deaths towards a majority of non-communicable deaths.^1–4^ However, not so much is known about the heterogeneity in such causes of death (e.g. are individuals dying from an increasingly varied set of causes?). Yet, exploration of how heterogeneous mortality profiles are is important both for theoretical and practical reasons. On the one hand, such information is needed to fully understand the biological and social drivers of morbimortality and to develop better conceptual and explanatory models. On the other hand, cause of death (CoD) heterogeneity is a central marker of populations’ health, informing about the predictability of death—a key ingredient for elaborating successful policies aimed at improving health and increasing longevity.

Several attempts have been made to document how diverse a given CoD profile is.^8–14^ More specifically, these studies define a ‘CoD diversity index’ to investigate whether deaths are highly concentrated in a limited set of causes or are widely scattered along the CoD classification list. For instance, focusing on a group of 15 low-mortality countries, Bergeron *et al*.^11^ report that, between 1994 and 2017, CoD diversity has increased as a consequence of the reductions in the share of cardiovascular deaths and the increase in deaths attributable to mental and behavioural disorders and diseases of the nervous system. A similar approach has been followed by Calazans and Permanyer^14^ to explore CoD diversity trends around the globe between 1990 and 2019. Unfortunately, those CoD diversity studies (i) fail to consider the extent of similarity/dissimilarity that might exist among pairs of causes of death, and (ii) have not quantified the contribution of each cause to overall CoD diversity levels. Imagine we were comparing two hypothetical mortality profiles, A and B, with three equally numerous causes of death only. In society A, one-third of deaths are attributable to interpersonal violence, one-third to transport injuries and the last third to unintentional injuries. In society B, one-third of deaths are due to Alzheimer’s disease, one-third to HIV/AIDS and one-third to interpersonal violence. Whereas CoD diversity indices would judge both societies to be equally diverse (in both cases, the share of deaths is equally split among three different causes of death), there are strong reasons to argue that the CoD profile is much more heterogeneous in society B than in society A. In A, all CoDs are ‘external’ and similar to each other, but in B some causes are ‘external’, some are ‘communicable’ and some are ‘non-communicable’. The main aim of this paper is to expand currently existing CoD diversity indicators by introducing measures that (i) are also sensitive to the similarity that might exist between the causes of death one is working with, and (ii) allow assessing the contribution of each cause to overall CoD heterogeneity levels.

## Measuring inequality in causes of death

Assume one is working with a set of k mutually exclusive causes of death. The (life table) share of deaths attributable to cause c will be denoted as $p_{c}$, so $\sum_{c}p_{c}=1$. The indicator proposed in this paper is defined as:

$$I=\sum_{i=1}^{k}\sum_{j=1}^{k}d_{ij}p_{i}p_{j}$$

where $d_{ij}\geq 0$ measures the degree of dissimilarity between causes of death i and j, with $d_{ii}=0$ for all i. The proposed I indicator measures the average expected dissimilarity between any two causes of death, so it will be referred to as an indicator of CoD inequality. Formally, this indicator shares a common structure with the well-known Gini coefficient frequently used to measure income inequality, which is defined as the average expected difference between individuals’ income levels (i.e. in that setting, $d_{ij}=\left|x_{i}-x_{j}\right|$, where $x_{i},x_{j}$ measure the income levels of individuals i and j). This substantively differs from current CoD diversity measures, which disregard the extent of (dis)similarity among pairs of causes. Interestingly, whenever all causes of death are assumed to be equally dissimilar with respect to each other (i.e. $d_{ij}$ takes the same value across all pairs of causes of death), CoD inequality becomes the diversity index proposed by Simpson,^15^ which is a well-known diversity measure—henceforth denoted as S—that, in this context, measures the likelihood that two randomly chosen deaths are attributable to different causes (Supplementary Material Section 2.1, available as Supplementary data at *IJE* online). Thus, our measure of CoD inequality can be seen as an extension of existing diversity measures. By definition, I attains the lowest value of 0 whenever all individuals die from the same cause, and increasingly higher values when individuals die from an increasingly variegated set of causes. When $d_{ij}$ does not change across CoD pairs, the measure is maximized whenever deaths are uniformly distributed across causes. In the three-causes example presented above, societies A and B are equally diverse according to S (and other traditional diversity measures). However, B should be more unequal than A according to I owing to the high similarity among CoDs in the latter’s mortality profile.

### Dissimilarities among causes of death

**Figure 1.**
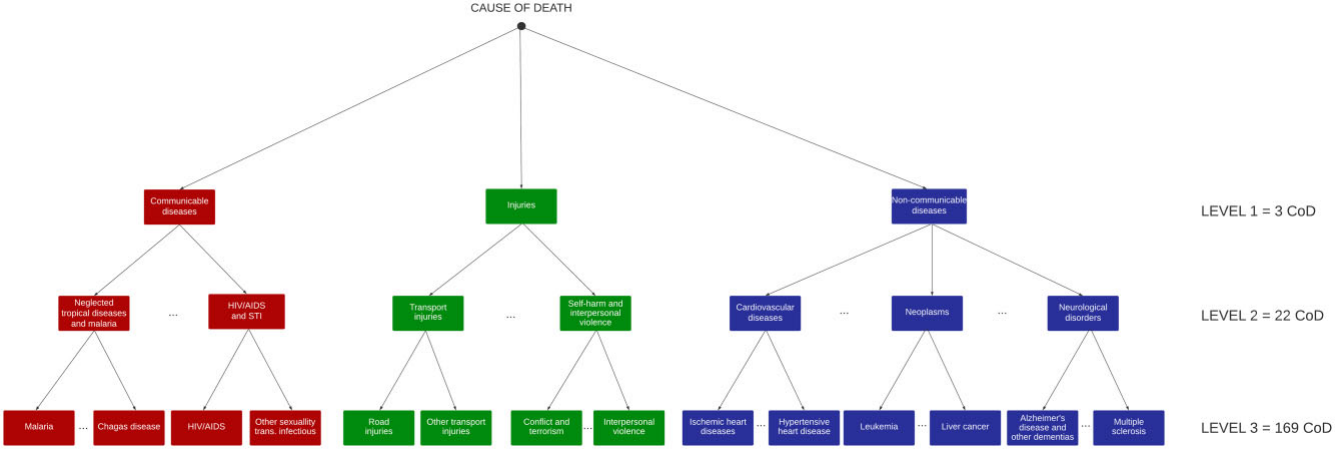
Schematic representation of the cause-of-death (CoD) classification tree used in the Global Burden of Disease project

There are many ways of measuring the degree of (dis)similarity among causes of death. Here we take advantage of the tree-like structures often used to classify causes of death, which start from a very general level and become increasingly granular at subsequent levels. In Figure 1, we illustrate the three-level tree-like structure of the CoD classification used by the Global Burden of Disease (GBD) project, which will be applied in the empirical application of this paper (the list of all causes of death can be found in Supplementary Material Section 1, available as Supplementary data at *IJE* online) and which is compatible with the different International Classification of Diseases (ICD) schemes. The first level is composed of three groups of causes of death: (i) communicable diseases, (ii) non-communicable diseases and (iii) injuries. The second level is composed of 22 groups, and the third level is composed of 167 groups. However, 34 categories in the third level had no cause of death over time, and were therefore excluded from the analysis. A fourth level of disaggregation is still available, but since there were many categories with zero deaths, we limited our analyses to the first three disaggregation levels.

Given any two causes of death i and j, the dissimilarity function $d_{ij}$ will be defined as the (normalized) length of the shortest path linking the two causes in the tree-like structure used to classify the different CoDs. Thus, $d_{ij}$ will take a value of 0 whenever both causes belong to the same group at level 3, a value of 1/3 whenever they belong to different groups at level 3 but to the same group at level 2 (e.g. ‘Ischaemic heart disease’ and ‘Hypertensive heart disease’), a value of 2/3 whenever they belong to different groups at level 2 but to the same group at level 1 (e.g. ‘Leukaemia’ and ‘Ischaemic heart disease’) and a value of 1 whenever they belong to different groups at level 1 (e.g. ‘Alzheimer’s disease’ and ‘Interpersonal Violence’). Choosing values of $d_{ij}$ between 0 and 1, we ensure that the values of our CoD inequality index are also bounded between 0 and 1. This simple and parsimonious approach to assess dissimilarity can be easily adapted to other tree-like classification schemes, not only in the context of epidemiology but in many other settings as well (e.g. industrial or job classifications in economics, taxonomy in biology and so on).

### Cause-specific decompositions

A useful feature of the inequality and diversity indices presented here is that they can be easily broken down by causes of death. In this way, one can assess how much each cause separately contributed to the observed CoD heterogeneity levels. Using the same notation as above, one can easily check that our measure of CoD inequality can be written as:

$$I=\sum_{c=1}^{k}\sum_{i=1}^{k}d_{ci}p_{c}p_{i}=\sum_{c=1}^{k}p_{c}\left[\sum_{i=1}^{k}d_{ci}p_{i}\right]=\;\sum_{c=1}^{k}p_{c}I_{c}=\sum_{c=1}^{k}C_{c}$$

Thus, the contribution of cause c to overall inequality levels ($C_{c}$) equals $p_{c}I_{c}$, where $I_{c}$ is the average distance between cause c and the remaining causes (i.e. $I_{c}=\sum_{i}d_{ci}p_{i}$). In the particular case where all $d_{ij}$ take the same value for all i≠j (i.e. all causes of death are assumed to be equally dissimilar vis-à-vis each other), then we obtain the diversity index S. When this happens, the contribution of cause c to overall diversity can be simply written as $p_{c}(1-p_{c})$ (see Supplementary Material Section 2.2, available as Supplementary data at *IJE* online).

## Empirical illustration

Using data from the GBD project [https://vizhub.healthdata.org/gbd-results/], we estimate the CoD inequality (*I*) and CoD diversity measures (S) between 1990 and 2019, for women and men separately, for 15 low-mortality countries (Australia, Austria, Belgium, Canada, Denmark, Finland, France, Germany, Japan, The Netherlands, Spain, Sweden, Switzerland, the UK and the USA). We also generate 95% uncertainty intervals (UIs) for the CoD inequality and diversity measures, taking advantage of the UIs reported by the GBD project for the mortality proportions for each of the causes of death (Supplementary Material Section 2.3, available as Supplementary data at *IJE* online).

**Figure 2.**
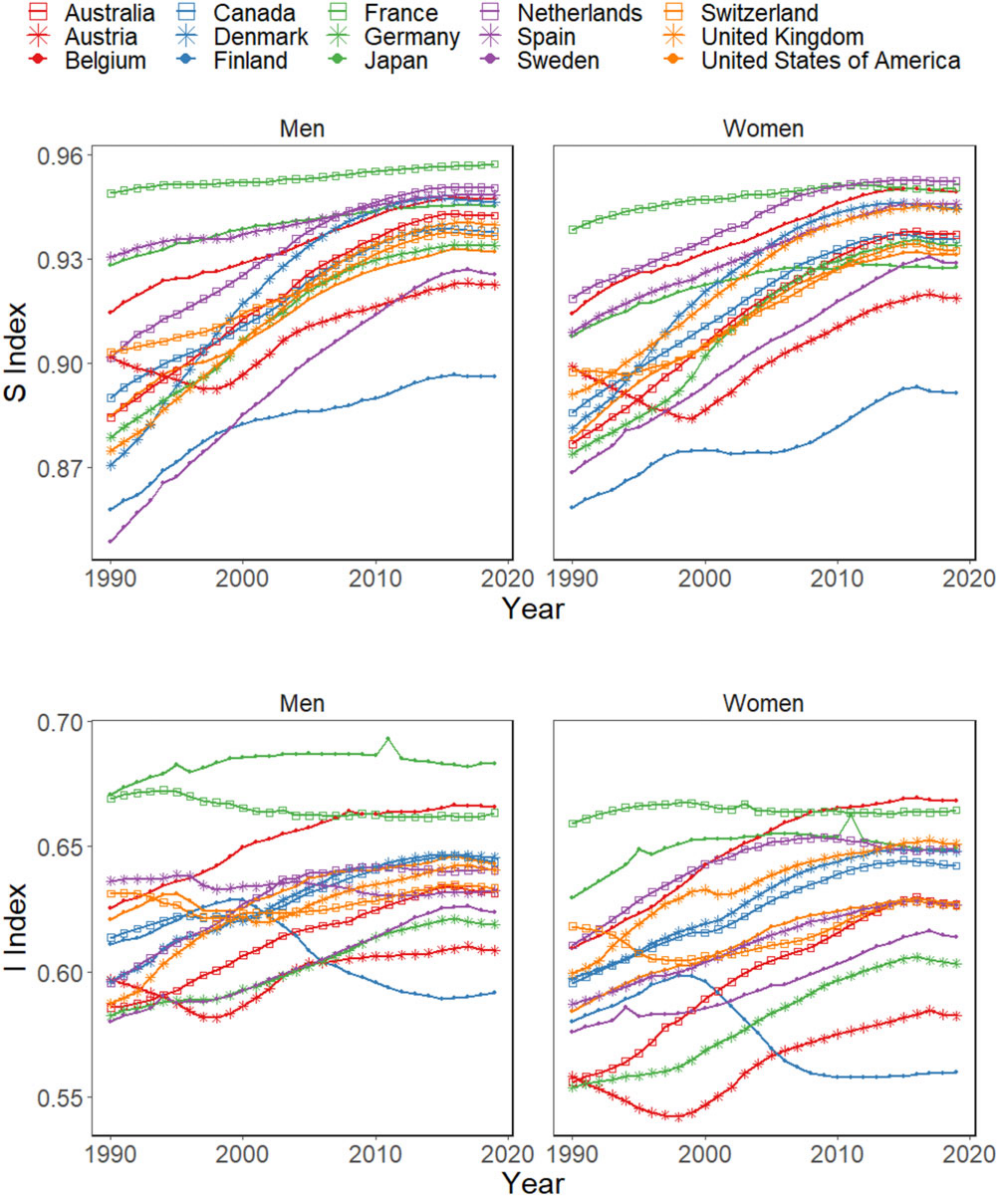
Trends in cause-of-death diversity (S) and inequality (I) indices between 1990 and 2019 in 15 select low-mortality countries for women and men. Source: Authors’ elaboration based on Global Burden of Disease/Institute for Health Metrics and Evaluation (GBD/IHME)

All countries show an increase in S between 1990 and 2019, but with different trends: many of them increase significantly (e.g. Sweden and Belgium), others increase mildly (e.g. France) and others follow non-monotonic trajectories (e.g. Austria and Finland). CoD inequality generally increases over time across countries and sex, except for the cases of Finland (both sexes) and Spanish and French males (see Figure 2 and Tables 1, 2). Over time, one can observe some spikes in the I and S trajectories, which are attributable to exogenous shocks such as the 2011 earth and seaquakes in Japan. The spikes in CoD inequality are more accentuated than in CoD diversity because of the greater dissimilarity between the exogenous shock causes and the remaining causes observed in the corresponding CoD profiles.

**Table 1.** Cause-of-death diversity index (S) in 15 select low-mortality countries, men and women (1990 and 2019)

	1990	2019	Difference
Men			
Australia	0.884 (0.867–0.902)	0.942 (0.935–0.950)	0.058*
Austria	0.902 (0.887–0.917)	0.923 (0.907–0.938)	0.021
Belgium	0.915 (0.903–0.926)	0.947 (0.940–0.954)	0.033*
Canada	0.890 (0.874–0.907)	0.938 (0.928–0.948)	0.048*
Denmark	0.871 (0.849–0.893)	0.946 (0.939–0.954)	0.076*
Finland	0.858 (0.833–0.883)	0.896 (0.871–0.922)	0.038
France	0.949 (0.944–0.954)	0.957 (0.954–0.960)	0.008*
Germany	0.879 (0.859–0.898)	0.934 (0.922–0.946)	0.055*
Japan	0.928 (0.923–0.933)	0.945 (0.940–0.950)	0.017*
The Netherlands	0.901 (0.887–0.916)	0.950 (0.945–0.956)	0.049*
Spain	0.931 (0.923–0.939)	0.948 (0.943–0.954)	0.018*
Sweden	0.849 (0.823–0.875)	0.926 (0.913–0.938)	0.077*
Switzerland	0.903 (0.886–0.922)	0.937 (0.926–0.947)	0.033*
UK	0.875 (0.862–0.888)	0.940 (0.935–0.945)	0.065*
USA	0.885 (0.873–0.896)	0.932 (0.925–0.939)	0.047*
Women			
Australia	0.877 (0.861–0.893)	0.937 (0.935–0.939)	0.060*
Austria	0.899 (0.883–0.915)	0.919 (0.911–0.926)	0.020
Belgium	0.914 (0.903–0.926)	0.949 (0.948–0.950)	0.035*
Canada	0.886 (0.874–0.898)	0.936 (0.935–0.936)	0.050*
Denmark	0.881 (0.862–0.901)	0.945 (0.943–0.946)	0.064*
Finland	0.858 (0.834–0.883)	0.891 (0.874–0.909)	0.033
France	0.938 (0.935–0.941)	0.951 (0.942–0.959)	0.012*
Germany	0.874 (0.854–0.893)	0.934 (0.929–0.939)	0.060*
Japan	0.908 (0.904–0.912)	0.928 (0.901–0.954)	0.020
The Netherlands	0.919 (0.910–0.927)	0.952 (0.951–0.953)	0.034*
Spain	0.909 (0.899–0.919)	0.946 (0.942–0.950)	0.037*
Sweden	0.869 (0.849–0.889)	0.929 (0.924–0.933)	0.060*
Switzerland	0.897 (0.883–0.912)	0.932 (0.932–0.932)	0.035*
UK	0.891 (0.879–0.903)	0.944 (0.942–0.946)	0.053*
USA	0.878 (0.869–0.887)	0.931 (0.929–0.934)	0.053*

Source: Authors’ elaboration based on Global Burden of Disease/Institute for Health Metrics and Evaluation (GBD/IHME); 95% uncertainty interval in parentheses.

* The difference in the index between 1990 and 2019 is statistically significant at a 5% uncertainty level.

**Table 2.** Cause-of-death inequality index (I) in 15 select low-mortality countries, men and women (1990 and 2019)

	1990	2019	Difference
Men			
Australia	0.586 (0.573–0.599)	0.632 (0.626–0.637)	0.046*
Austria	0.597 (0.590–0.605)	0.609 (0.599–0.618)	0.011
Belgium	0.626 (0.617–0.634)	0.666 (0.662–0.669)	0.040*
Canada	0.614 (0.602–0.625)	0.644 (0.637–0.651)	0.030*
Denmark	0.596 (0.580–0.613)	0.646 (0.641–0.651)	0.049*
Finland	0.611 (0.594–0.628)	0.592 (0.573–0.611)	–0.019*
France	0.669 (0.665–0.674)	0.663 (0.663–0.664)	–0.006*
Germany	0.583 (0.570–0.595)	0.619 (0.610–0.628)	0.037*
Japan	0.671 (0.669–0.672)	0.683 (0.678–0.688)	0.012*
The Netherlands	0.596 (0.584–0.607)	0.641 (0.637–0.644)	0.045*
Spain	0.636 (0.630–0.643)	0.633 (0.628–0.637)	–0.004
Sweden	0.580 (0.561–0.600)	0.624 (0.614–0.634)	0.043*
Switzerland	0.631 (0.619–0.643)	0.634 (0.628–0.639)	0.002
UK	0.587 (0.579–0.596)	0.641 (0.639–0.643)	0.053*
USA	0.621 (0.616–0.626)	0.643 (0.642–0.645)	0.022*
Women			
Australia	0.556 (0.539–0.574)	0.627 (0.625–0.630)	0.071*
Austria	0.558 (0.549–0.568)	0.583 (0.573–0.592)	0.024*
Belgium	0.609 (0.597–0.621)	0.668 (0.667–0.669)	0.059*
Canada	0.596 (0.583–0.609)	0.642 (0.640–0.645)	0.047*
Denmark	0.597 (0.581–0.614)	0.648 (0.644–0.652)	0.051*
Finland	0.580 (0.560–0.602)	0.560 (0.542–0.578)	–0.020
France	0.659 (0.652–0.666)	0.665 (0.660–0.669)	0.005
Germany	0.554 (0.537–0.572)	0.603 (0.595–0.612)	0.049*
Japan	0.629 (0.628–0.631)	0.649 (0.632–0.667)	0.020*
The Netherlands	0.611 (0.601–0.621)	0.649 (0.648–0.650)	0.038*
Spain	0.587 (0.575–0.600)	0.627 (0.626–0.628)	0.039*
Sweden	0.576 (0.559–0.594)	0.614 (0.608–0.620)	0.038*
Switzerland	0.618 (0.607–0.630)	0.626 (0.626–0.627)	0.008
UK	0.599 (0.589–0.610)	0.651 (0.651–0.651)	0.052*
USA	0.584 (0.576–0.593)	0.628 (0.627–0.629)	0.043*

Source: Authors’ elaboration based on Global Burden of Disease/Institute for Health Metrics and Evaluation (GBD/IHME); 95% uncertainty interval in parentheses.

* The difference in the index between 1990 and 2019 is statistically significant at a 5% uncertainty level.

In several countries, I and S increase simultaneously. However, the trends in I and S do not necessarily go in the same direction. In the countries where diversity increases and inequality declines, the causes of death are becoming increasingly unpredictable, but the similarity between such causes is increasingly high. As a result, the overall association between both indicators is positive but not particularly strong. In 2019, Kendall’s rank correlation coefficient between I and S (an indicator ranging between -1 and 1 akin to Pearson’s correlation coefficient but measuring the ordinal association between two measured quantities) equals 0.50 among men and 0.52 among women.

As shown in Tables 1 and 2, the values of S and I tend to be higher among men, but this is not always the case (particularly in 2019).

**Figure 3.**
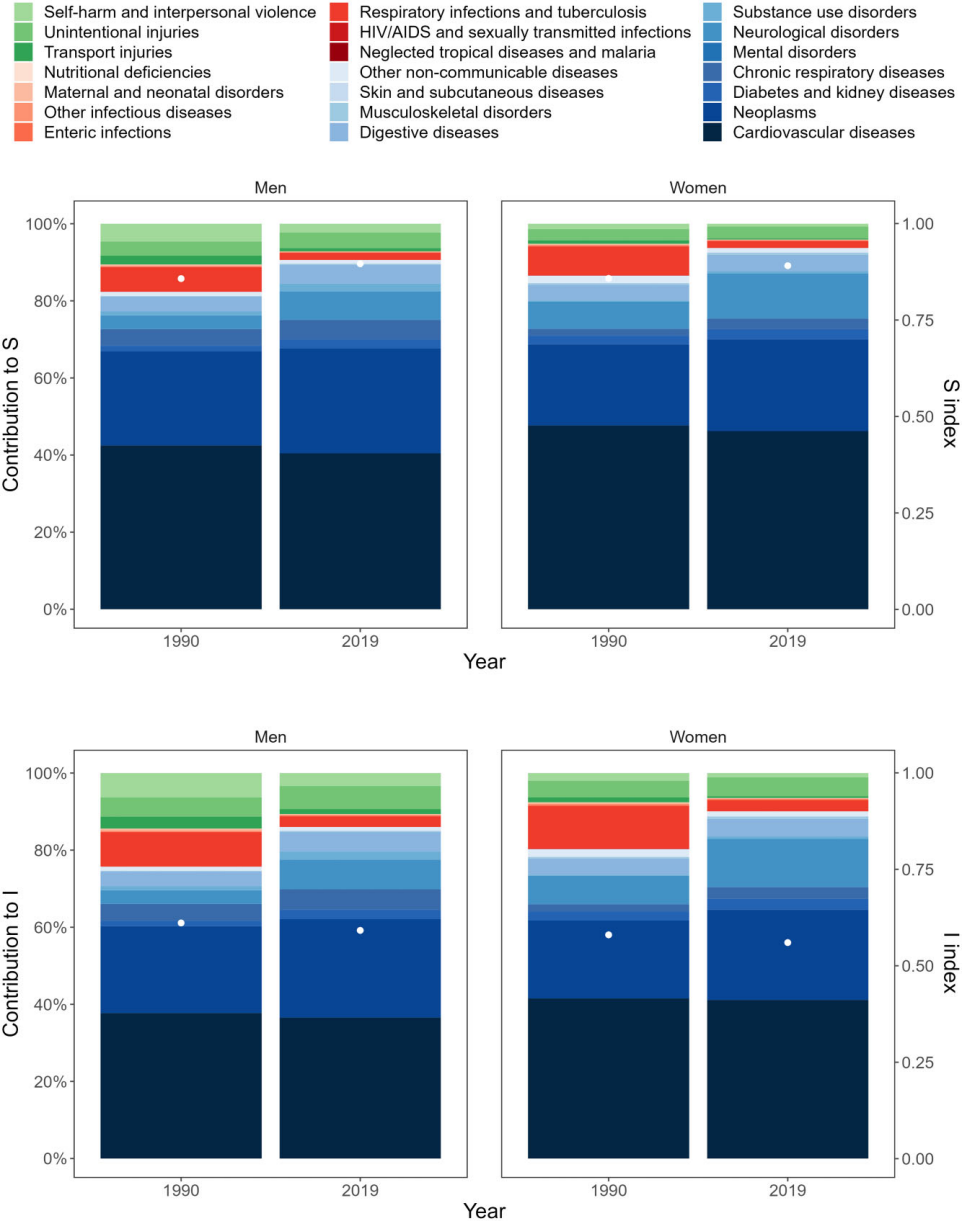
Levels and cause-specific decompositions of the cause-of-death diversity (S) and inequality (I) indices in Finland for men and women (1990 and 2019). Note: the scale of the cause-specific decomposition contributions is shown on the left-hand vertical axis, and the indices scale is on the right-hand axis. In order to facilitate visualization, the 133 causes of death in level 3 have been aggregated in the graph into their corresponding level 2 categories. Source: Authors’ elaboration based on Global Burden of Disease/Institute for Health Metrics and Evaluation (GBD/IHME)

The CoD diversity and inequality indices can be broken down by cause. Figure 3 shows those decompositions for Finland (results for the remaining countries are shown in the Supplementary Material Section 3, Figures S1–S14, available as Supplementary data at *IJE* online). Between 1990 and 2019, CoD inequality decreased from 0.580 (0.560 to 0.602) to 0.560 (0.542 to 0.578) for women and from 0.611 (0.594 to 0.628) to 0.592 (0.573 to 0.611) for men. On the other hand, CoD diversity increased from 0.858 (0.834 to 0.883) to 0.891 (0.874 to 0.909) for women and from 0.858 (0.833 to 0.883) to 0.896 (0.871 to 0.922) for men in the same period. CoD diversity and inequality are explained mainly by non-communicable causes (coloured in blue shades). In 1990, the 106 causes of death that make up this group contributed to explain 80.25% and 86.51% of the levels in I and S for women and 75.70% and 82.33% for men, respectively. Over time, the contribution of non-communicable causes becomes more pronounced for both indicators. Among non-communicable causes, cardiovascular diseases make the largest contributions to both indicators—despite the fact such contributions slightly decrease over time. Finland follows a similar pattern to the other countries included in the analysis, with declines in the contribution of cardiovascular diseases and an increase in the other non-communicable causes (see Supplementary Material Section 3, Figures S1–S14, available as Supplementary data at *IJE* online).

The contributions of communicable causes (coloured in red shades) and injuries (green shades) to changes in both indicators are significantly lower than the contributions of non-communicable causes—and they tend to decrease over time. In 1990, the 46 communicable causes explained 12.16% of CoD inequality for women and 9.89% for men. In 2019, these values fell to 3.49% and 3.37% for women and men, respectively. This runs counter to the experience of other countries like Belgium, Denmark or The Netherlands, which have seen increases in the contribution of communicable causes to CoD inequality (see Supplementary Material Section 3 Figures, available as Supplementary data at *IJE* online).

The 10 causes in the injury group explain 7.69% of CoD inequality levels in 1990 and 6.46% in 2019 for women, and those contributions go from 14.41% in 1990 to 10.63% in 2019 for men. In contrast, the contribution of injuries to CoD inequality increased over time in some other countries (e.g. Belgium, The Netherlands). Notably, the contributions of communicable causes and injuries to CoD diversity are smaller than the contributions to CoD inequality. This is attributable to the higher-than-average dissimilarity between those causes and the rest of causes which concentrate the majority of deaths in the analysed countries (the non-communicable ones; see Figure 1).

## Discussion

Since the introduction of the Epidemiological Transition theory,^1^ many studies have documented the evolution of the CoD structure in different world regions.^4–7^^,^^16–20^ We already know much about the main causes of death over time (e.g. which have generally shifted from ‘communicable’ to ‘non-communicable’ deaths). However, our knowledge about the heterogeneity in the causes leading to death is still incipient and limited. Earlier studies have proposed measures of CoD diversity^8–14^ but are insensitive to the degree of similarity between the causes one is working with. This study presents a new class of population health metrics (the so-called ‘CoD inequality measures’) that generalizes the previous indicators, incorporating a simple distance function between causes of death which can be easily adapted to many disparate settings where the categories are tree-structured. In this way, we avoid treating causes of death as a purely categorical, structureless variable, by introducing some further information reflecting the commonalities or disparities that might exist between them. In addition, the indicators are decomposable by cause.

CoD inequality indicators capture novel information that cannot be revealed through diversity measures alone. Indeed, in our empirical example we find several countries where CoD diversity and inequality measures go in opposite directions. For instance, among men in Finland, France and Spain and among women in Finland, increases in CoD diversity go in tandem with declines in CoD inequality. In those settings, the specific causes from which individuals die become less predictable over time, but the ‘average similarity’ between such causes becomes increasingly high—a phenomenon that is attributable to the increasing concentration of main causes of death within the same branches and sub-branches of the CoD classification tree (see Figure 1), where they share increasingly similar aetiologies. For several countries, CoD diversity and inequality increase simultaneously. This pattern can be associated with the emergence of neurodegenerative disorders, like Alzheimer’s disease and other types of dementia, which gradually replace the deaths attributable to the cardiovascular system owing to the success of the so-called ‘cardiovascular revolution’^4^ (see Supplementary Material Section 3, Figures S1–S14, available as Supplementary data at *IJE* online). In some countries (e.g. Belgium, Denmark), the increasing contribution of communicable deaths (which are ‘far away’ from the majority of non-communicable deaths in the CoD classification tree), lead to vigorous increases in CoD inequality (see Figure 2). Altogether, our findings indicate that CoD diversity and inequality indicators offer complementary information about the heterogeneity in mortality profiles, so they should be analysed alongside other metrics commonly used to assess population health, such as life expectancy and lifespan inequality.^11^^,^^14^^,^^21–23^

In our empirical illustration, CoD inequality and diversity indicators tend to be higher among men than among women. This happens because men’s CoD profile is more heterogeneous than women’s due to a higher prevalence of mortality from external causes among the former. This means that the causes from which men die tend to be more dissimilar and unpredictable than those prevailing among women.

Indicators of CoD heterogeneity are very useful markers of populations’ health. Greater heterogeneity could imply lower predictability and more dissimilarity among the causes from which individuals die. Consequently, efforts to reduce mortality would have to address a more variegated set of causes, thus rendering attempts at further increasing longevity potentially more ineffective.^11^^,^^14^ The information generated by the CoD heterogeneity indicators and the cause-specific decompositions here presented provides thus valuable insights into the elaboration of effective health policies and the promotion of social and preventive medicine.

Our proposed CoD heterogeneity measures have some limitations. To begin with, there are many alternative ways of measuring the degree of dissimilarity among causes of death. We have followed a simple, flexible and parsimonious approach, but other more sophisticated methods are certainly possible. One could, for instance, use disease genes to identify the common genetic origin of many diseases^24^ or define aetiologically-based distance functions. In addition, our findings are contingent on the ways in which the CoD classification trees are constructed. Alternative CoD groupings could lead to inconsistent assessments. For this reason, analyses involving comparisons across space and time should all be based on the same classification scheme (as in our empirical illustration). The quality of the data source we are working with could potentially lead to some sort of bias. It is well-known that GBD estimates are often modelled on the basis of weak and imperfect data, particularly in low- and middle-income countries.^25^ Therefore, we have restricted our attention to a relatively small group of high-income countries where the quality of the data is unlikely to be problematic, and leave for future research the exploration of CoD inequality dynamics in other world regions. Last, the values of heterogeneity measures (like our CoD inequality indicators) alone might not be very telling. This is why the decomposition techniques proposed here—which inform users about the contribution of each cause of death to overall heterogeneity levels—are so useful and informative. Overall, the new CoD inequality and diversity indicators and the corresponding cause-specific decompositions are attractive methodologies for studying the heterogeneity in populations’ health that complement currently existing approaches.

## Ethics approval

Not required.

## Supplementary Material

dyae016_Supplementary_Data

## Data Availability

The scripts generated during the current study are available in the Git-hub repository [https://github.com/healinproject/CoD-Inequality]. We conducted our analyses using the open-source statistical software R (version R-4.1.0).
