# Supplementary material for: On the measurement of cause of death inequality
Source: Int J Epidemiol. 2024 Feb 14;53(2):dyae016. doi: 10.1093/ije/dyae016 (PMC10873278; doi:10.1093/ije/dyae016)
Supplement: dyae016_Supplementary_Data [file dyae016_supplementary_data.docx]

**SUPPLEMENTARY MATERIAL**

**Table of contents**

[SECTION 1. CAUSES OF DEATH CLASSIFICATION 2](#_Toc156468658)

[SECTION 2. MATHEMATICAL APPENDIX 7](#_Toc156468659)

[**2.1. Simpson’s Diversity Index (**$S$**)** 7](#_Toc156468660)

[**2.2. Cause-specific decomposition for** $S$ 7](#_Toc156468661)

[**2.3.** **Uncertainty analysis** 8](#_Toc156468662)

[SECTION 3. RESULTS 8](#_Toc156468663)

# **SECTION 1. CAUSES OF DEATH CLASSIFICATION**

We used the same causes of deaths classification as the Global Burden of Disease (GBD) Project 2019. The Global Burden of Disease (GBD) project aggregates the causes of deaths in a tree-like structure. The first level is composed of three groups of causes of death: (1) communicable diseases, (2) non-communicable diseases, and (3) injuries. The second level is composed of 21 groups, and the third level is composed of 167 groups. However, 34 categories in the third level had no cause of death overtime and were therefore excluded from the analysis. A fourth level of disaggregation is still available, but since there were many categories with zero deaths, we preferred to work with only three disaggregation levels. The causes that make up the first three levels of aggregation are listed in Table S1.

### Table S1 – Causes of death classification, according Global Burden of Disease Study 2019

| Cause ID | Cause Name | Level |
| --- | --- | --- |
| 295 | **Communicable, maternal, neonatal, and nutritional diseases** | 1 |
| 955 | *HIV/AIDS and sexually transmitted infections* | 2 |
| 298 | HIV/AIDS | 3 |
| 393 | Sexually transmitted infections excluding HIV | 3 |
| 956 | *Respiratory infections and tuberculosis* | 2 |
| 297 | Tuberculosis | 3 |
| 322 | Lower respiratory infections | 3 |
| 328 | Upper respiratory infections | 3 |
| 329 | Otitis media | 3 |
| 957 | *Enteric infections* | 2 |
| 302 | Diarrheal diseases | 3 |
| 958 | Typhoid and paratyphoid | 3 |
| 959 | Invasive Non-typhoidal Salmonella (iNTS) | 3 |
| 321 | Other intestinal infectious diseases | 3 |
| 344 | *Neglected tropical diseases and malaria* | 2 |
| 345 | Malaria | 3 |
| 346 | Chagas disease | 3 |
| 347 | Leishmaniasis | 3 |
| 350 | African trypanosomiasis | 3 |
| 351 | Schistosomiasis | 3 |
| 352 | Cysticercosis | 3 |
| 353 | Cystic echinococcosis | 3 |
| 354 | Lymphatic filariasis* | 3 |
| 355 | Onchocerciasis* | 3 |
| 356 | Trachoma* | 3 |
| 357 | Dengue | 3 |
| 358 | Yellow fever | 3 |
| 359 | Rabies | 3 |
| 360 | Intestinal nematode infections | 3 |
| 364 | Food-borne trematodiases* | 3 |
| 405 | Leprosy* | 3 |
| 843 | Ebola | 3 |
| 935 | Zika vírus | 3 |
| 936 | Guinea worm disease* | 3 |
| 365 | Other neglected tropical diseases | 3 |
| 961 | *Other infectious diseases* | 2 |
| 332 | Meningitis | 3 |
| 337 | Encephalitis | 3 |
| 338 | Diphtheria | 3 |
| 339 | Whooping cough | 3 |
| 340 | Tetanus | 3 |
| 341 | Measles | 3 |
| 342 | Varicella and herpes zoster | 3 |
| 400 | Acute hepatites | 3 |
| 408 | Other unspecified infectious diseases | 3 |
| 962 | *Maternal and neonatal disorders* | 2 |
| 366 | Maternal disorders | 3 |
| 380 | Neonatal disorders | 3 |
| 386 | *Nutritional deficiencies* | 2 |
| 387 | Protein-energy malnutrition | 3 |
| 388 | Iodine deficiency* | 3 |
| 389 | Vitamin A deficiency* | 3 |
| 390 | Dietary iron deficiency* | 3 |
| 391 | Other nutritional deficiencies | 3 |
| 409 | **Non-communicable diseases** | 1 |
| 410 | *Neoplasms* | 2 |
| 444 | Lip and oral cavity câncer | 3 |
| 447 | Nasopharynx câncer | 3 |
| 450 | Other pharynx câncer | 3 |
| 411 | Esophageal câncer | 3 |
| 414 | Stomach câncer | 3 |
| 441 | Colon and rectum câncer | 3 |
| 417 | Liver câncer | 3 |
| 453 | Gallbladder and biliary tract cancer | 3 |
| 456 | Pancreatic câncer | 3 |
| 423 | Larynx câncer | 3 |
| 426 | Tracheal, bronchus, and lung cancer | 3 |
| 459 | Malignant skin melanoma | 3 |
| 462 | Non-melanoma skin câncer | 3 |
| 429 | Breast câncer | 3 |
| 432 | Cervical câncer | 3 |
| 435 | Uterine câncer | 3 |
| 465 | Ovarian câncer | 3 |
| 438 | Prostate câncer | 3 |
| 468 | Testicular câncer | 3 |
| 471 | Kidney câncer | 3 |
| 474 | Bladder câncer | 3 |
| 477 | Brain and central nervous system cancer | 3 |
| 480 | Thyroid câncer | 3 |
| 483 | Mesothelioma | 3 |
| 484 | Hodgkin lymphoma | 3 |
| 485 | Non-Hodgkin lymphoma | 3 |
| 486 | Multiple myeloma | 3 |
| 487 | Leukemia | 3 |
| 1022 | Other malignant neoplasms | 3 |
| 490 | Other neoplasms | 3 |
| 491 | *Cardiovascular diseases* | 2 |
| 492 | Rheumatic heart disease | 3 |
| 493 | Ischemic heart disease | 3 |
| 494 | Stroke | 3 |
| 498 | Hypertensive heart disease | 3 |
| 504 | Non-rheumatic valvular heart disease | 3 |
| 499 | Cardiomyopathy and myocarditis | 3 |
| 500 | Atrial fibrillation and flutter | 3 |
| 501 | Aortic aneurysm | 3 |
| 502 | Peripheral artery disease | 3 |
| 503 | Endocarditis | 3 |
| 1023 | Other cardiovascular and circulatory diseases | 3 |
| 508 | *Chronic respiratory diseases* | 2 |
| 509 | Chronic obstructive pulmonary disease | 3 |
| 510 | Pneumoconiosis | 3 |
| 515 | Asthma | 3 |
| 516 | Interstitial lung disease and pulmonary sarcoidosis | 3 |
| 520 | Other chronic respiratory diseases | 3 |
| 526 | *Digestive diseases* | 2 |
| 521 | Cirrhosis and other chronic liver diseases | 3 |
| 992 | Upper digestive system diseases | 3 |
| 529 | Appendicitis | 3 |
| 530 | Paralytic ileus and intestinal obstruction | 3 |
| 531 | Inguinal, femoral, and abdominal hernia | 3 |
| 532 | Inflammatory bowel disease | 3 |
| 533 | Vascular intestinal disorders | 3 |
| 534 | Gallbladder and biliary diseases | 3 |
| 535 | Pancreatitis | 3 |
| 541 | Other digestive diseases | 3 |
| 542 | *Neurological disorders* | 2 |
| 543 | Alzheimer's disease and other dementias | 3 |
| 544 | Parkinson's disease | 3 |
| 545 | Idiopathic epilepsy | 3 |
| 546 | Multiple sclerosis | 3 |
| 554 | Motor neuron disease | 3 |
| 972 | Headache disorders* | 3 |
| 557 | Other neurological disorders | 3 |
| 558 | *Mental disorders* | 2 |
| 559 | Schizophrenia* | 3 |
| 567 | Depressive disorders | 3 |
| 570 | Bipolar disorder* | 3 |
| 571 | Anxiety disorders* | 3 |
| 572 | Eating disorders | 3 |
| 575 | Autism spectrum disorders* | 3 |
| 578 | Attention-deficit/hyperactivity disorder* | 3 |
| 579 | Conduct disorder* | 3 |
| 582 | Idiopathic developmental intellectual disability* | 3 |
| 585 | Other mental disorders* | 3 |
| 973 | *Substance use disorders* | 2 |
| 560 | Alcohol use disorders | 3 |
| 561 | Drug use disorders | 3 |
| 974 | *Diabetes and kidney diseases* | 2 |
| 587 | Diabetes mellitus | 3 |
| 589 | Chronic kidney disease | 3 |
| 588 | Acute glomerulonephritis | 3 |
| 653 | *Skin and subcutaneous diseases* | 2 |
| 654 | Dermatitis* | 3 |
| 655 | Psoriasis* | 3 |
| 980 | Bacterial skin diseases | 3 |
| 658 | Scabies* | 3 |
| 659 | Fungal skin diseases* | 3 |
| 660 | Viral skin diseases* | 3 |
| 661 | Acne vulgaris* | 3 |
| 662 | Alopecia areata* | 3 |
| 663 | Pruritus* | 3 |
| 664 | Urticaria* | 3 |
| 665 | Decubitus ulcer | 3 |
| 668 | Other skin and subcutaneous diseases | 3 |
| 669 | *Sense organ diseases* | 2 |
| 981 | Blindness and vision loss* | 3 |
| 674 | Age-related and other hearing loss* | 3 |
| 679 | Other sense organ diseases* | 3 |
| 626 | *Musculoskeletal disorders* | 2 |
| 627 | Rheumatoid arthritis | 3 |
| 628 | Osteoarthritis* | 3 |
| 630 | Low back pain* | 3 |
| 631 | Neck pain* | 3 |
| 632 | Gout* | 3 |
| 639 | Other musculoskeletal disorders | 3 |
| 640 | *Other non-communicable diseases* | 2 |
| 641 | Congenital birth defects | 3 |
| 594 | Urinary diseases and male infertility | 3 |
| 603 | Gynecological diseases | 3 |
| 613 | Hemoglobinopathies and hemolytic anemias | 3 |
| 619 | Endocrine, metabolic, blood, and immune disorders | 3 |
| 680 | Oral disorders* | 3 |
| 686 | Sudden infant death syndrome | 3 |
| 687 | **Injuries** | 1 |
| 688 | *Transport injuries* | 2 |
| 689 | Road injuries | 3 |
| 695 | Other transport injuries | 3 |
| 696 | *Unintentional injuries* | 2 |
| 697 | Falls | 3 |
| 698 | Drowning | 3 |
| 699 | Fire, heat, and hot substances | 3 |
| 700 | Poisonings | 3 |
| 704 | Exposure to mechanical forces | 3 |
| 708 | Adverse effects of medical treatment | 3 |
| 709 | Animal contact | 3 |
| 712 | Foreign body | 3 |
| 842 | Environmental heat and cold exposure | 3 |
| 729 | Exposure to forces of nature | 3 |
| 716 | Other unintentional injuries | 3 |
| 717 | *Self-harm and interpersonal violence* | 2 |
| 718 | Self-harm | 3 |
| 724 | Interpersonal violence | 3 |
| 945 | Conflict and terrorism | 3 |
| 854 | Executions and police conflict | 3 |

Note: * Causes with no deaths

# **SECTION 2. MATHEMATICAL APPENDIX**

## **2.1.** **Simpson’s Diversity Index (**$\boldsymbol{S}$**)**

The Simpson’s Diversity Index ($S$) can be expressed as:

$$S=\sum_{i=1}^{k} \sum_{j\neq i} p_{i}p_{j}=1- \sum_{i=1}^{k} p_{i}^{2}$$

where $p_{i}$ is the share of deaths from cause $i$ taken from the life table, and $k$ is the number of causes of death. By construction, the sum of the different $p_{i}$ equals 1.

Assuming all deaths are classified in a list of mutually exclusive causes, $S$ is defined as the probability that two randomly chosen deaths are attributable to different causes. Lower values indicate that deaths are increasingly concentrated in fewer causes. In the limit, if all individuals died from the same cause, $S$ would equal zero. At the other extreme, higher values of $S$ indicate that the causes from which individuals die become increasingly diverse. The $S$ index is maximized when deaths are equally distributed across all possible causes (when this happens, $S$ equals $(k-1)/k$, where $k$ is the number of CoD – which corresponds to $20/21\cong0.95$ in our setting).

When calculating the $S$ index, we use the proportion of deaths by cause from the corresponding life tables (rather than the observed number of deaths) to render populations with different age structures comparable.

## **2.2. Cause-specific decomposition for** $\boldsymbol{S}$

When all $d_{ij}$ take the same value for all $i\neq j$ (say, $d_{ij}=1$), then one has that

$$I_{c}=\sum_{i=1}^{k} d_{ci}p_{i}=\sum_{i\neq c} p_{i}=1-p_{c}$$

because $d_{ii}=0$ for all $i$, and $\sum_{i} p_{i}=1$. In this setting

$$\mathcal{C}_{c}=p_{c}I_{c}=p_{c}(1-p_{c})$$

as we wanted to demonstrate.

## **Uncertainty analysis**

The GBD provides its mortality estimates with the 95% uncertainty interval. Thus, the proportion of deaths by cause in the life table will consider both the uncertainty in estimating the *dx* function and the uncertainty in estimating the proportions of deaths by causes before correction by age structure based on the life table.

Since neither the CoD Diversity Index (Simpson's Diversity Index) nor the CoD Inequality Index can take values lower than zero, we assume they follow a log-normal distribution, [[[Endnote #S1]]], with mean μ and standard deviation s:

$\mu=\log\left( \frac{m^{2}}{\sqrt{s^{2}+m^{2}}} \right) \mathrm{and} \sigma^{2}=\log\left( 1+\frac{s^{2}}{m^{2}} \right)$

Since $m$ is the mean and $s$ is the standard deviation of the normal distribution, we approximated the standard deviation by dividing the range of the 95% uncertainty interval by 3.92. We randomly drew 20,000 samples from these values, using Monte Carlo simulation techniques to obtain the uncertainty intervals for the indicators from the selected percentiles.

# **SECTION 3. RESULTS**

**
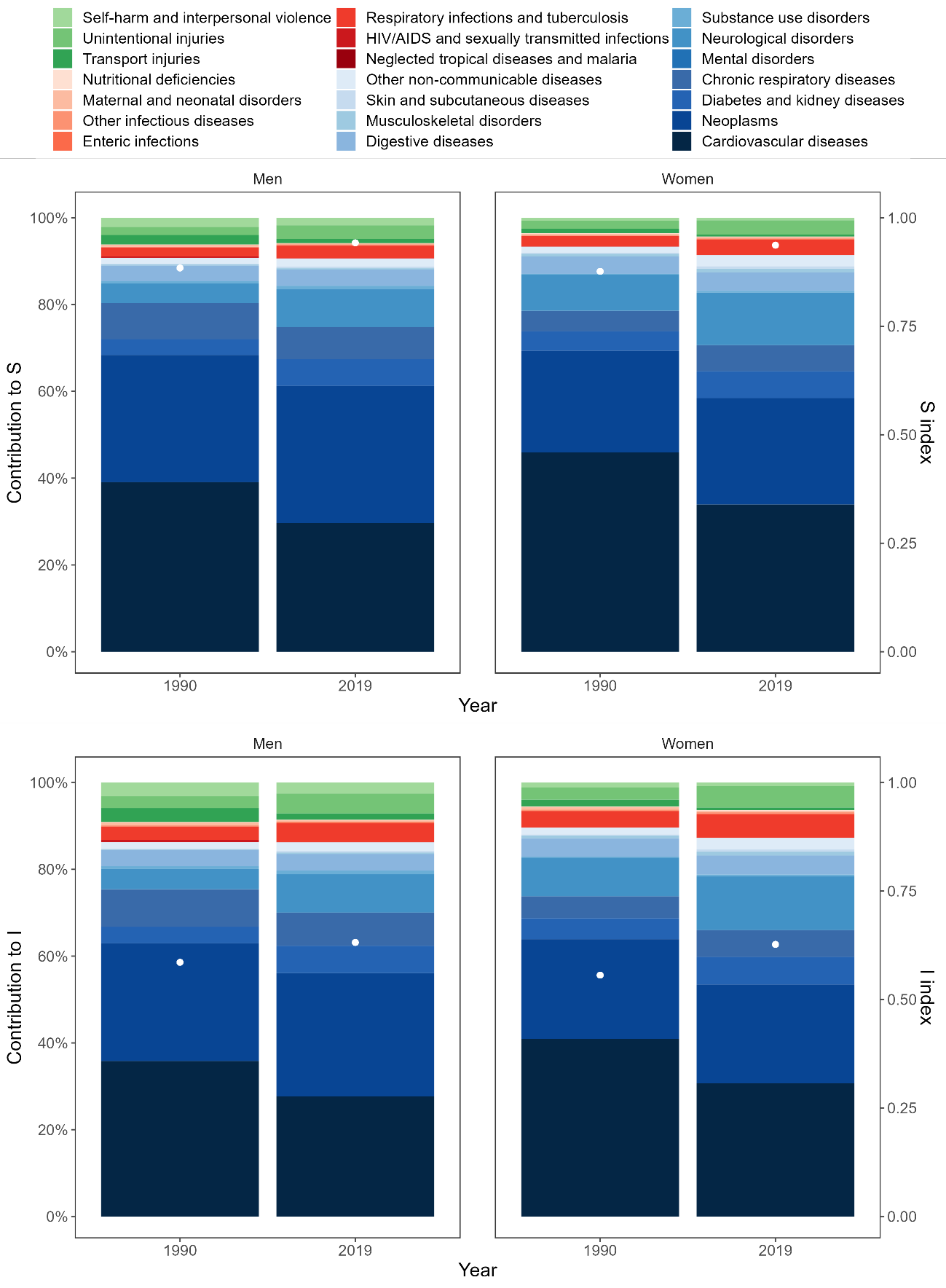
**

**Figure S1. Levels and cause-specific decompositions of the cause-of-death diversity (S) and inequality (I) indices in Australia for men and women (1990 and 2019).** Note: The scale of the cause-specific decomposition contributions is shown in the left-hand vertical axis, and the indices scale is in the right-hand one. In order to facilitate visualization, the 133 causes of death in level 3 have been aggregated in the graph into their corresponding level 2 categories. Source: Authors’ elaboration based on Global Burden of Disease/Institute for Health Metrics and Evaluation (GBD/IHME).

**
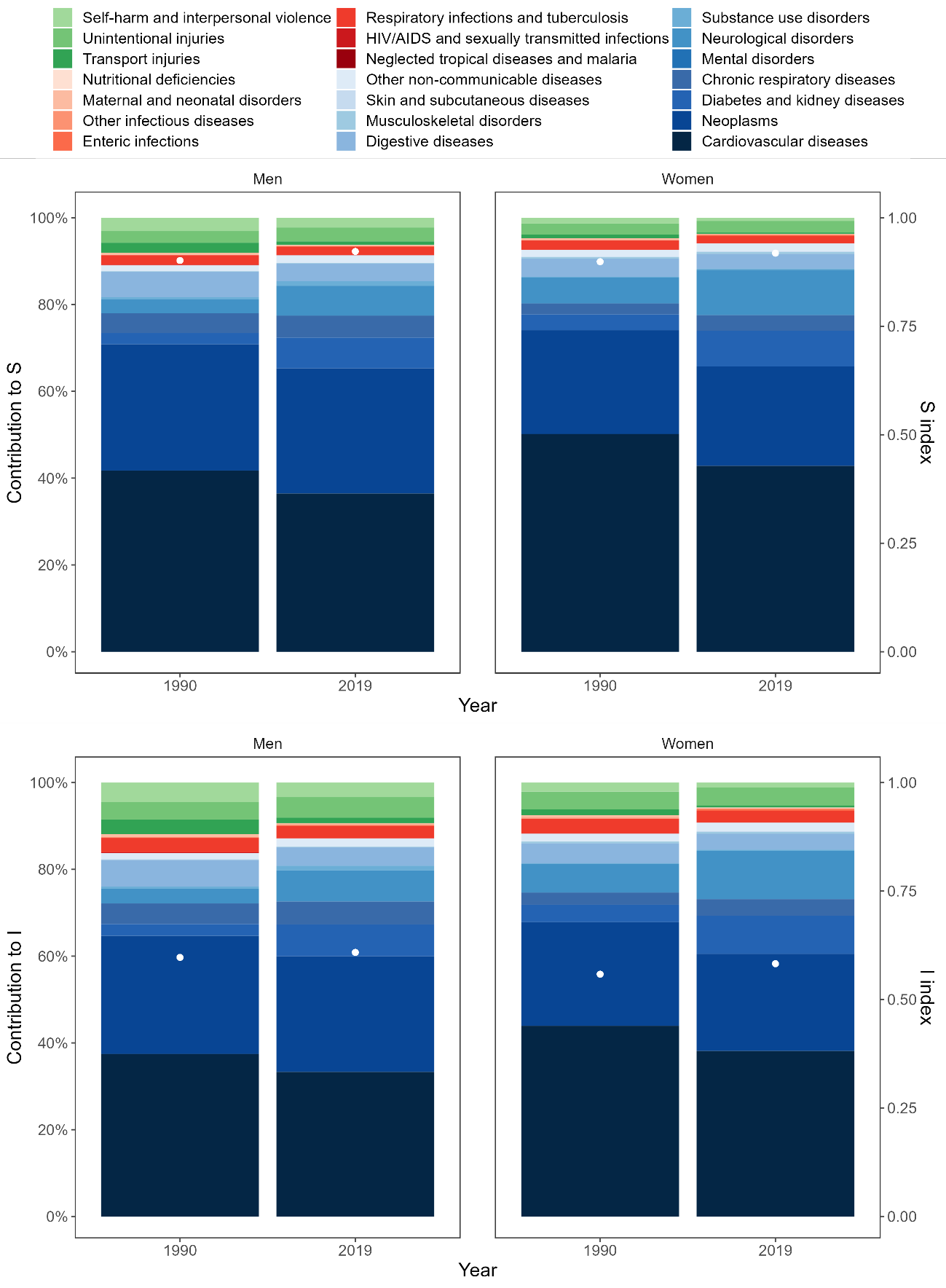
**

**Figure S2. Levels and cause-specific decompositions of the cause-of-death diversity (S) and inequality (I) indices in Austria for men and women (1990 and 2019).** Note: The scale of the cause-specific decomposition contributions is shown in the left-hand vertical axis, and the indices scale is in the right-hand one. In order to facilitate visualization, the 133 causes of death in level 3 have been aggregated in the graph into their corresponding level 2 categories. Source: Authors’ elaboration based on Global Burden of Disease/Institute for Health Metrics and Evaluation (GBD/IHME).

**
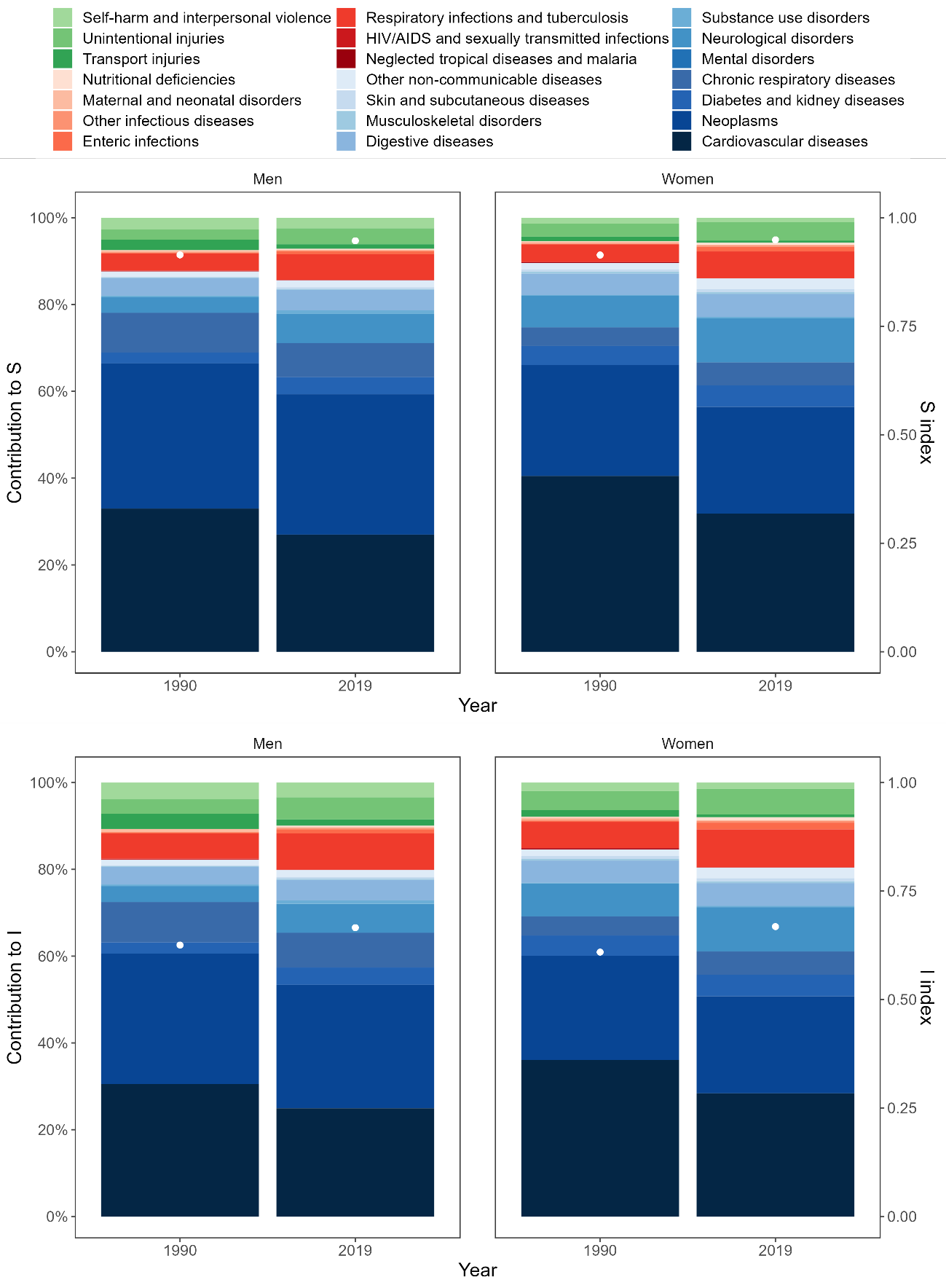
**

**Figure S3. Levels and cause-specific decompositions of the cause-of-death diversity (S) and inequality (I) indices in Belgium for men and women (1990 and 2019).** Note: The scale of the cause-specific decomposition contributions is shown in the left-hand vertical axis, and the indices scale is in the right-hand one. In order to facilitate visualization, the 133 causes of death in level 3 have been aggregated in the graph into their corresponding level 2 categories. Source: Authors’ elaboration based on Global Burden of Disease/Institute for Health Metrics and Evaluation (GBD/IHME).

**
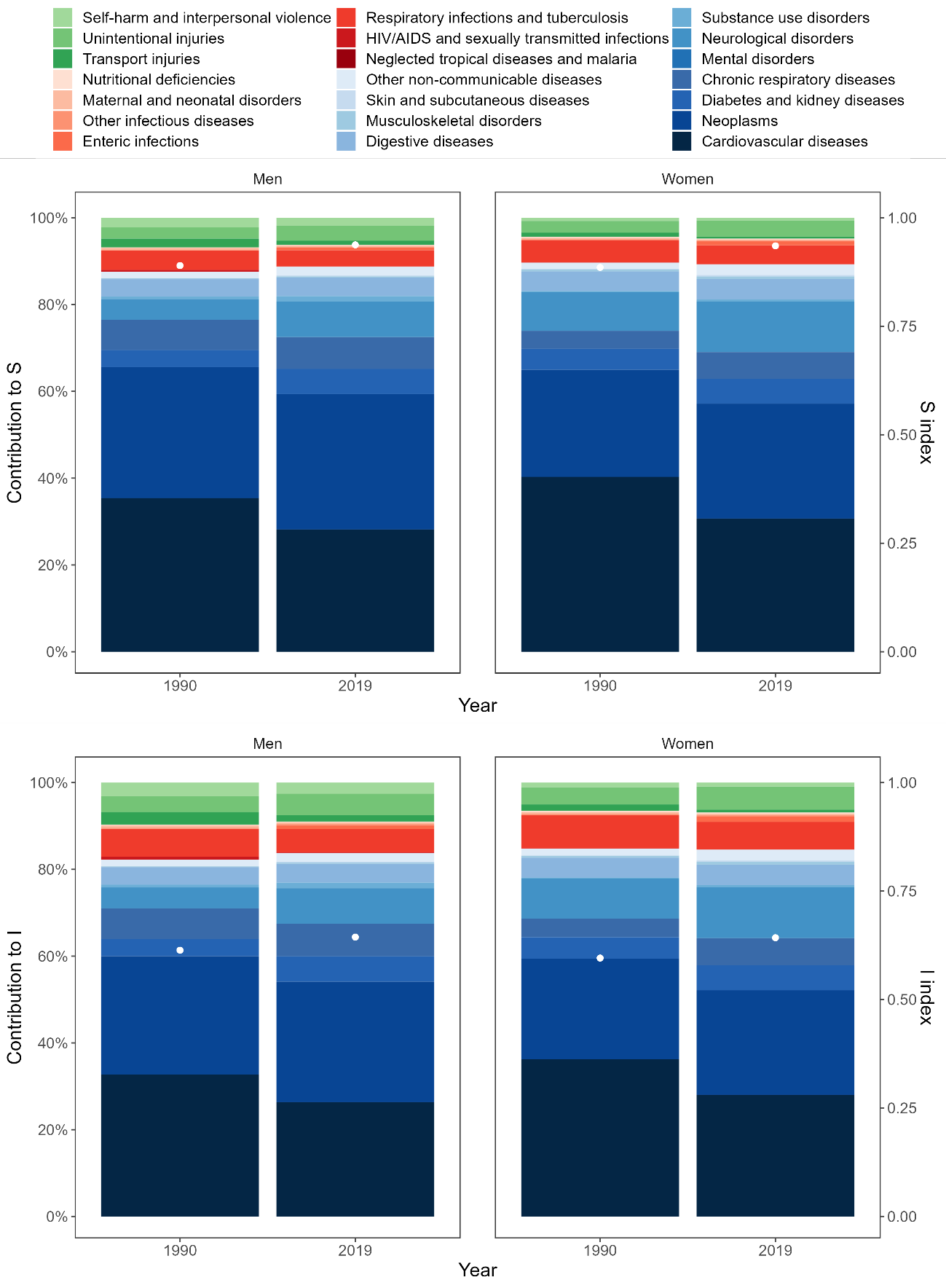
**

**Figure S4. Levels and cause-specific decompositions of the cause-of-death diversity (S) and inequality (I) indices in Canada for men and women (1990 and 2019).** Note: The scale of the cause-specific decomposition contributions is shown in the left-hand vertical axis, and the indices scale is in the right-hand one. In order to facilitate visualization, the 133 causes of death in level 3 have been aggregated in the graph into their corresponding level 2 categories. Source: Authors’ elaboration based on Global Burden of Disease/Institute for Health Metrics and Evaluation (GBD/IHME).

**
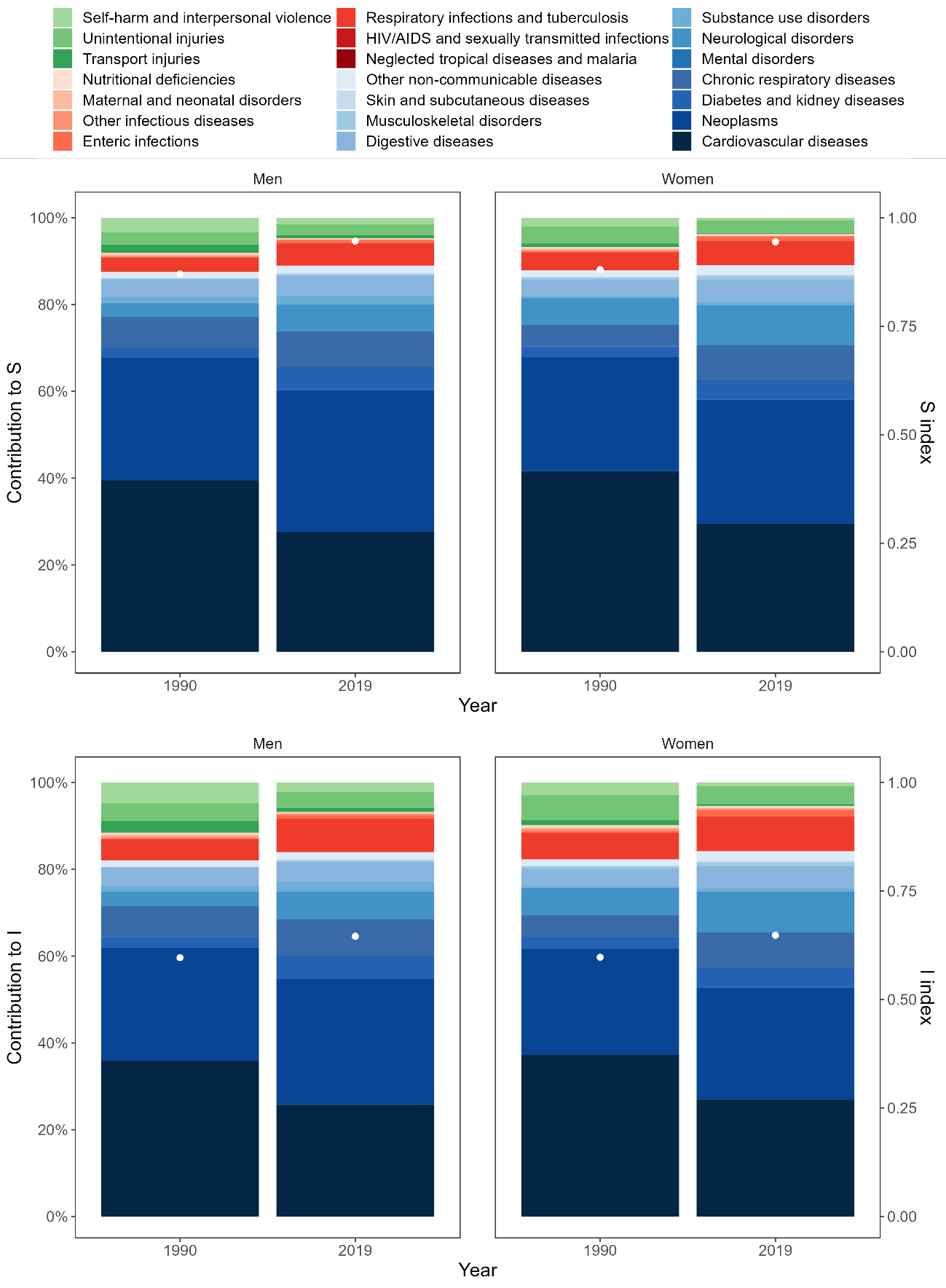
**

**Figure S5. Levels and cause-specific decompositions of the cause-of-death diversity (S) and inequality (I) indices in Denmark for men and women (1990 and 2019).** Note: The scale of the cause-specific decomposition contributions is shown in the left-hand vertical axis, and the indices scale is in the right-hand one. In order to facilitate visualization, the 133 causes of death in level 3 have been aggregated in the graph into their corresponding level 2 categories. Source: Authors’ elaboration based on Global Burden of Disease/Institute for Health Metrics and Evaluation (GBD/IHME).

**
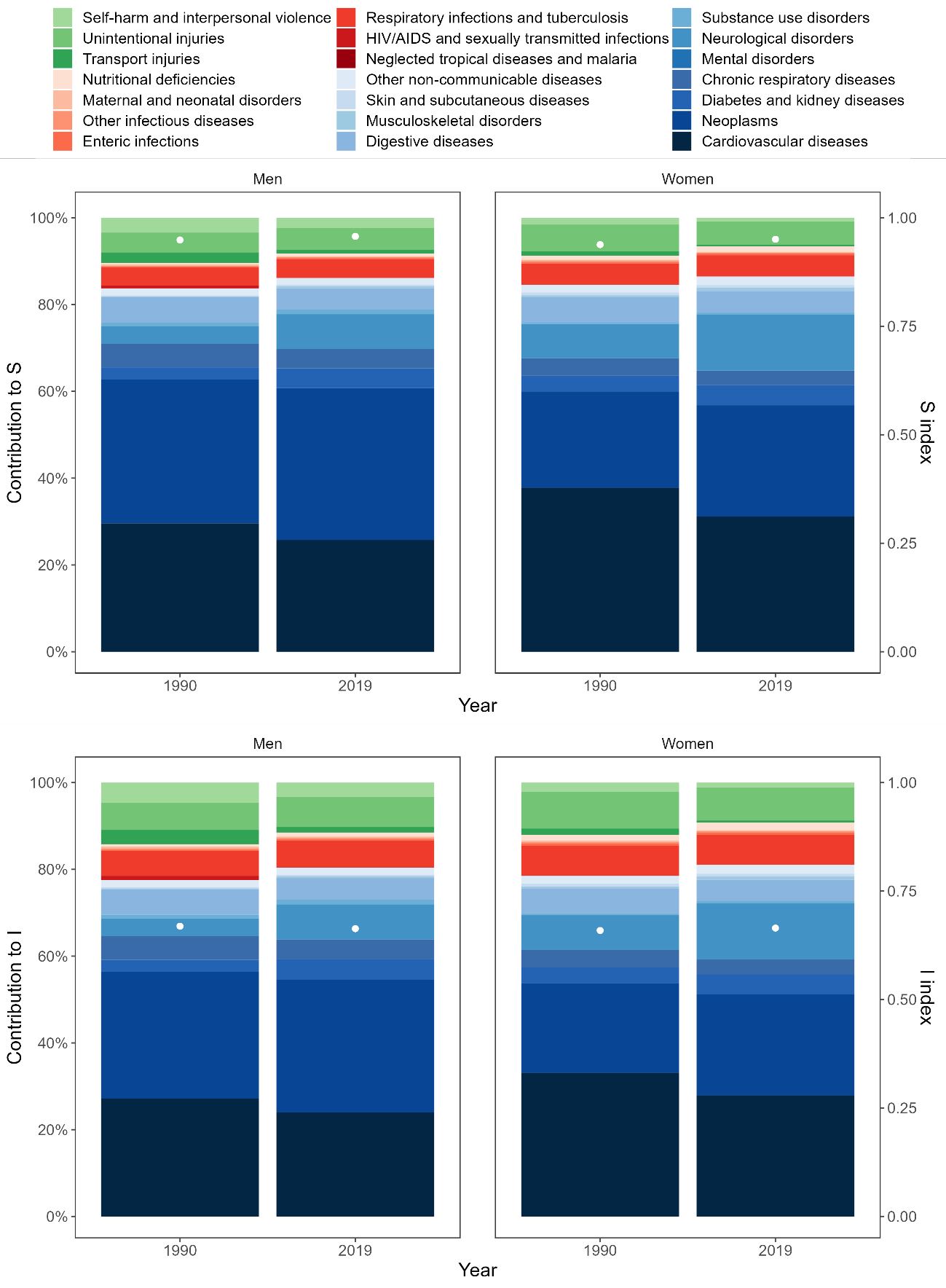
Figure S6. Levels and cause-specific decompositions of the cause-of-death diversity (S) and inequality (I) indices in France for men and women (1990 and 2019).** Note: The scale of the cause-specific decomposition contributions is shown in the left-hand vertical axis, and the indices scale is in the right-hand one. In order to facilitate visualization, the 133 causes of death in level 3 have been aggregated in the graph into their corresponding level 2 categories. Source: Authors’ elaboration based on Global Burden of Disease/Institute for Health Metrics and Evaluation (GBD/IHME).

**
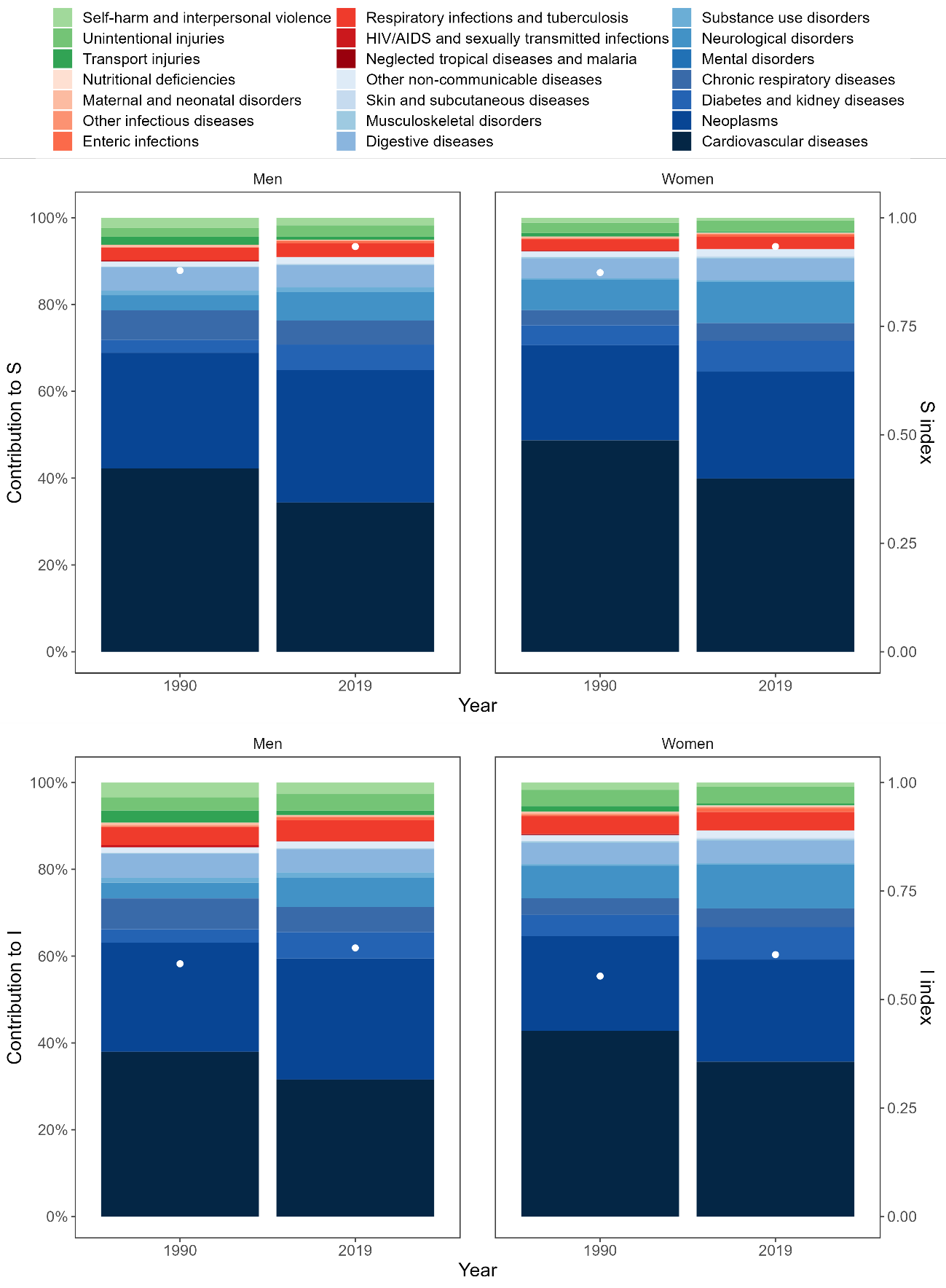
Figure S7. Levels and cause-specific decompositions of the cause-of-death diversity (S) and inequality (I) indices in Germany for men and women (1990 and 2019).** Note: The scale of the cause-specific decomposition contributions is shown in the left-hand vertical axis, and the indices scale is in the right-hand one. In order to facilitate visualization, the 133 causes of death in level 3 have been aggregated in the graph into their corresponding level 2 categories. Source: Authors’ elaboration based on Global Burden of Disease/Institute for Health Metrics and Evaluation (GBD/IHME).


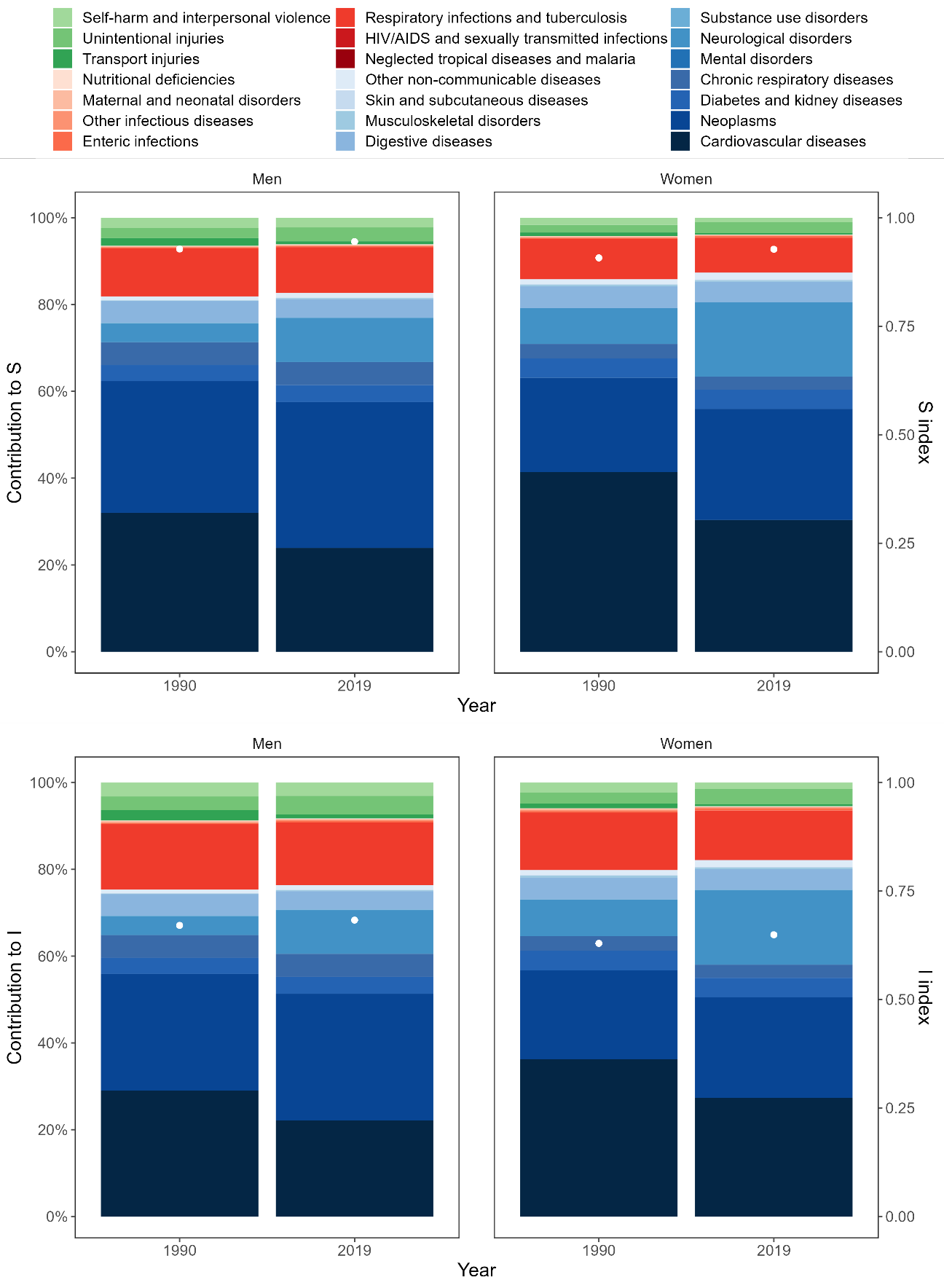


**Figure S8. Levels and cause-specific decompositions of the cause-of-death diversity (S) and inequality (I) indices in Japan for men and women (1990 and 2019).** Note: The scale of the cause-specific decomposition contributions is shown in the left-hand vertical axis, and the indices scale is in the right-hand one. In order to facilitate visualization, the 133 causes of death in level 3 have been aggregated in the graph into their corresponding level 2 categories. Source: Authors’ elaboration based on Global Burden of Disease/Institute for Health Metrics and Evaluation (GBD/IHME).

**
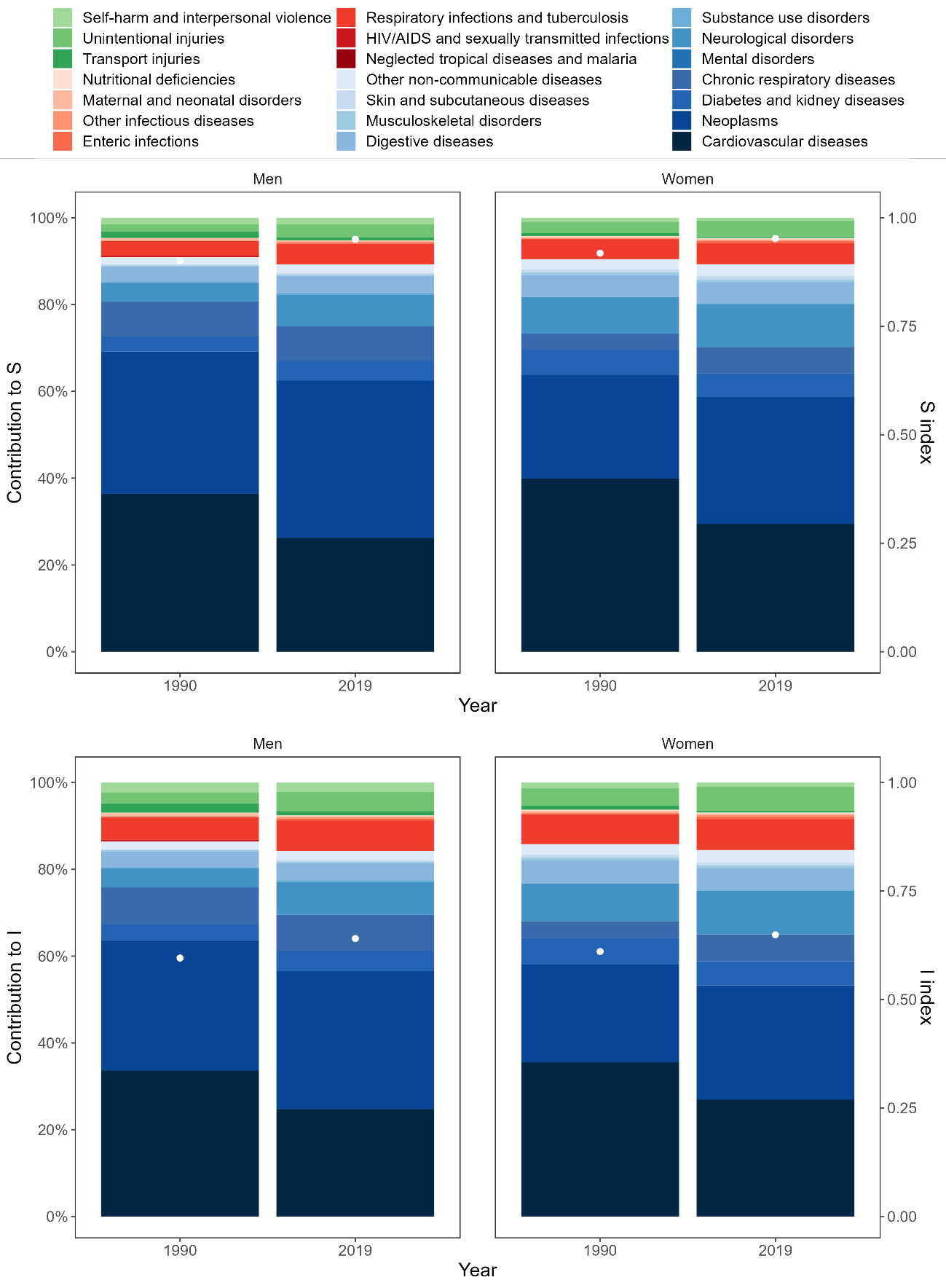
**

**Figure S9. Levels and cause-specific decompositions of the cause-of-death diversity (S) and inequality (I) indices in the Netherlands for men and women (1990 and 2019).** Note: The scale of the cause-specific decomposition contributions is shown in the left-hand vertical axis, and the indices scale is in the right-hand one. In order to facilitate visualization, the 133 causes of death in level 3 have been aggregated in the graph into their corresponding level 2 categories. Source: Authors’ elaboration based on Global Burden of Disease/Institute for Health Metrics and Evaluation (GBD/IHME).

**
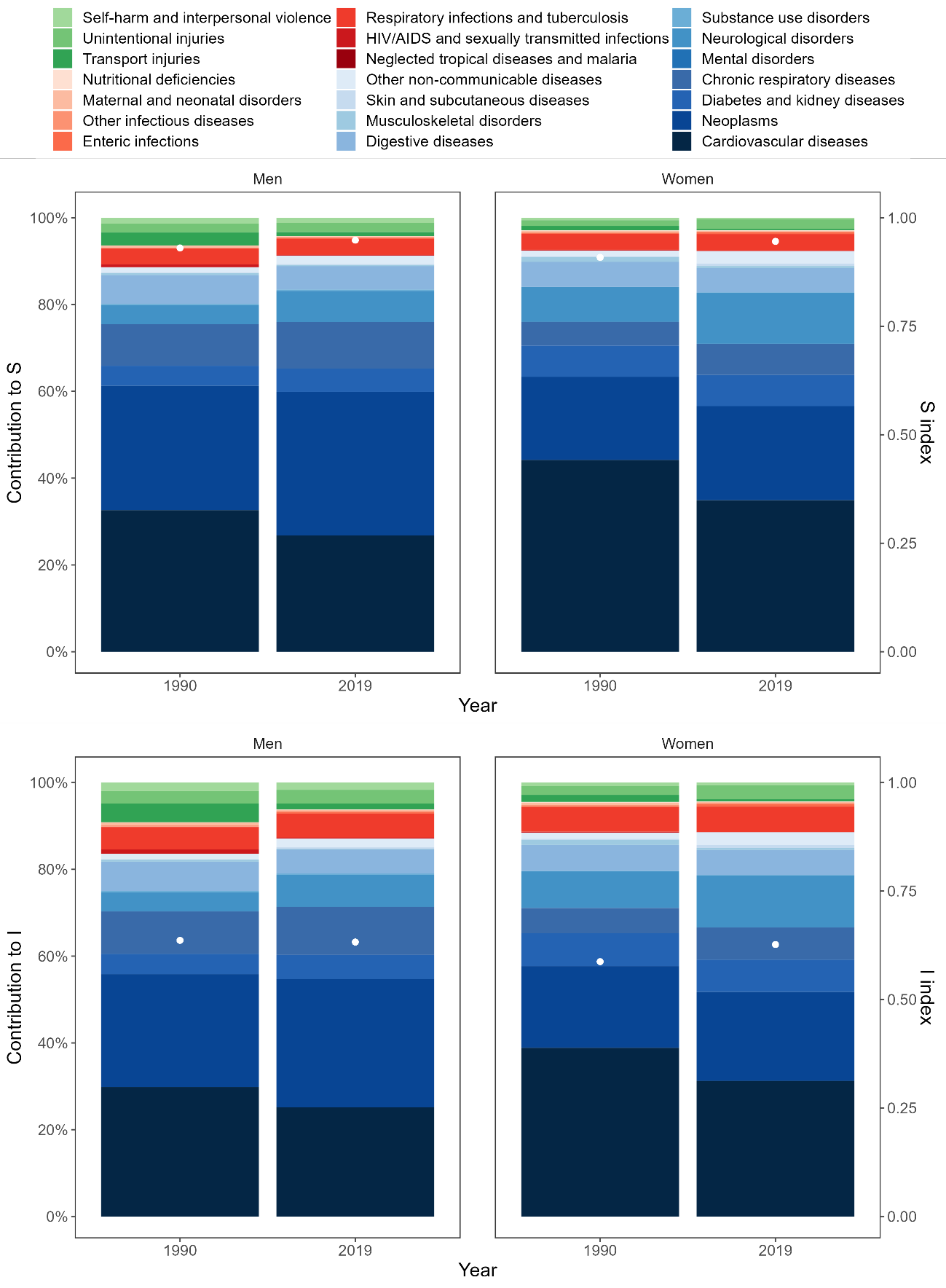
**

**Figure S10. Levels and cause-specific decompositions of the cause-of-death diversity (S) and inequality (I) indices in Spain for men and women (1990 and 2019).** Note: The scale of the cause-specific decomposition contributions is shown in the left-hand vertical axis, and the indices scale is in the right-hand one. In order to facilitate visualization, the 133 causes of death in level 3 have been aggregated in the graph into their corresponding level 2 categories. Source: Authors’ elaboration based on Global Burden of Disease/Institute for Health Metrics and Evaluation (GBD/IHME).

**
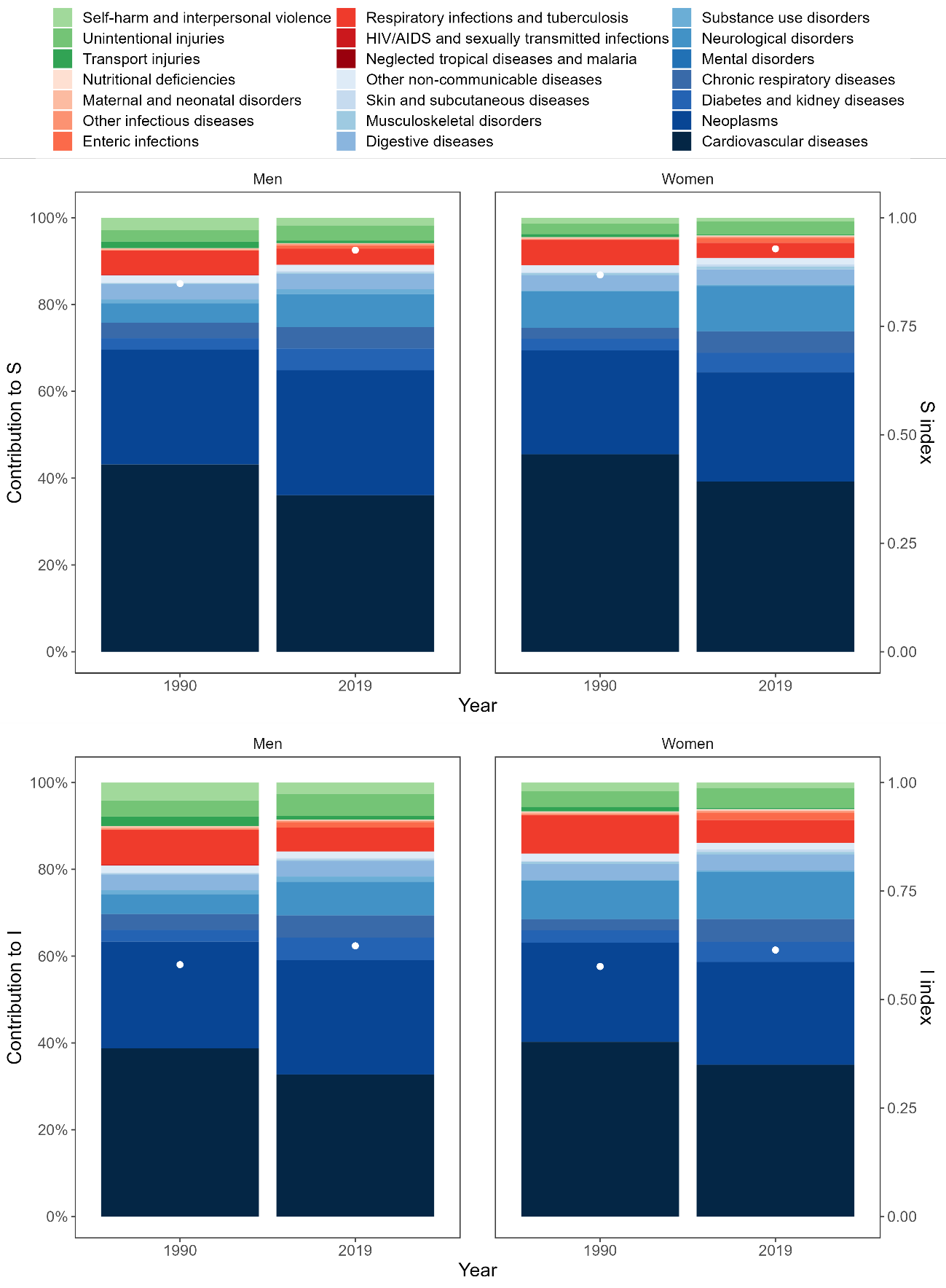
 Figure S11. Levels and cause-specific decompositions of the cause-of-death diversity (S) and inequality (I) indices in Sweden for men and women (1990 and 2019).** Note: The scale of the cause-specific decomposition contributions is shown in the left-hand vertical axis, and the indices scale is in the right-hand one. In order to facilitate visualization, the 133 causes of death in level 3 have been aggregated in the graph into their corresponding level 2 categories. Source: Authors’ elaboration based on Global Burden of Disease/Institute for Health Metrics and Evaluation (GBD/IHME).

**
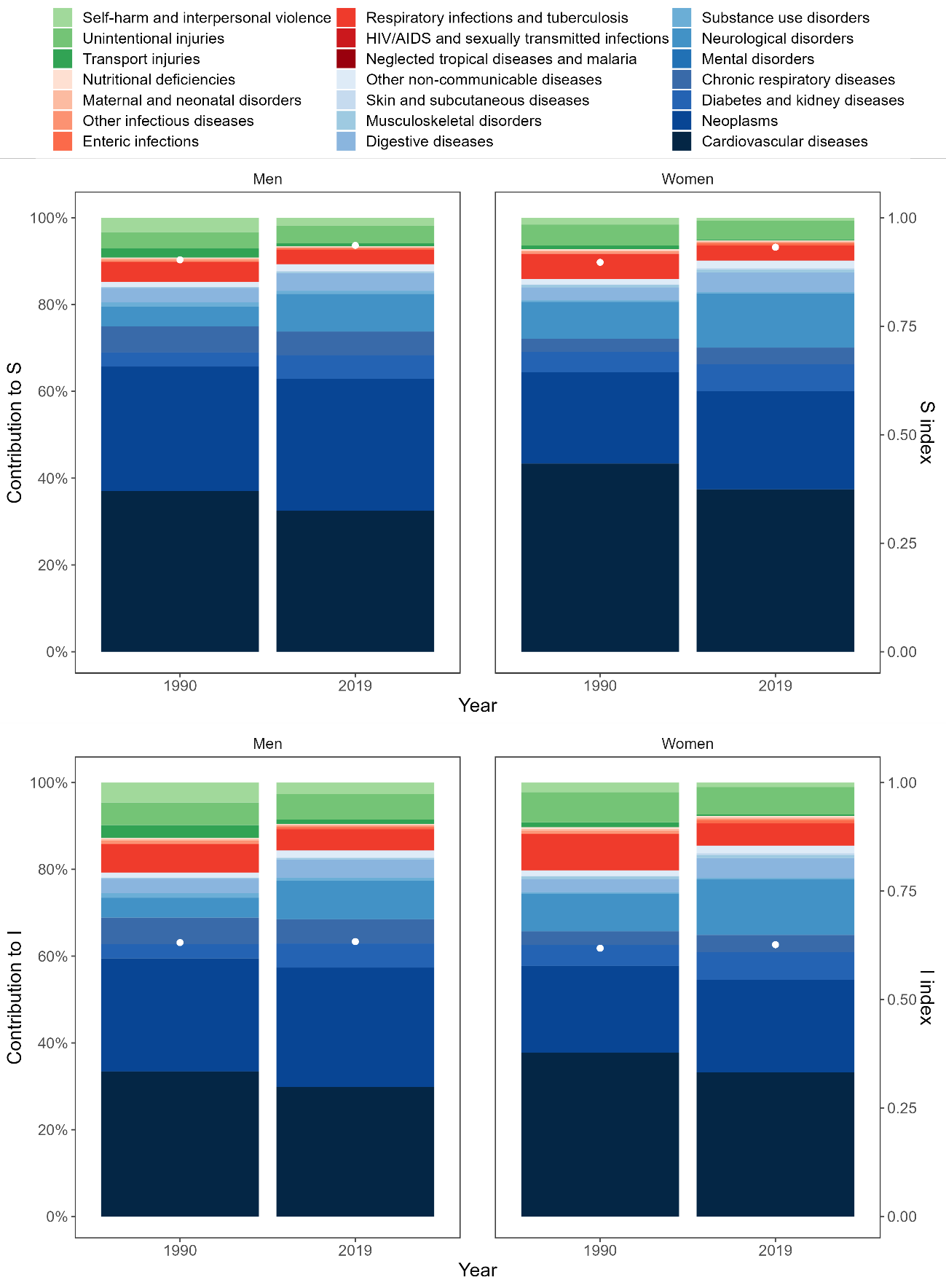
**

**Figure S12. Levels and cause-specific decompositions of the cause-of-death diversity (S) and inequality (I) indices in Switzerland for men and women (1990 and 2019).** Note: The scale of the cause-specific decomposition contributions is shown in the left-hand vertical axis, and the indices scale is in the right-hand one. In order to facilitate visualization, the 133 causes of death in level 3 have been aggregated in the graph into their corresponding level 2 categories. Source: Authors’ elaboration based on Global Burden of Disease/Institute for Health Metrics and Evaluation (GBD/IHME).

**
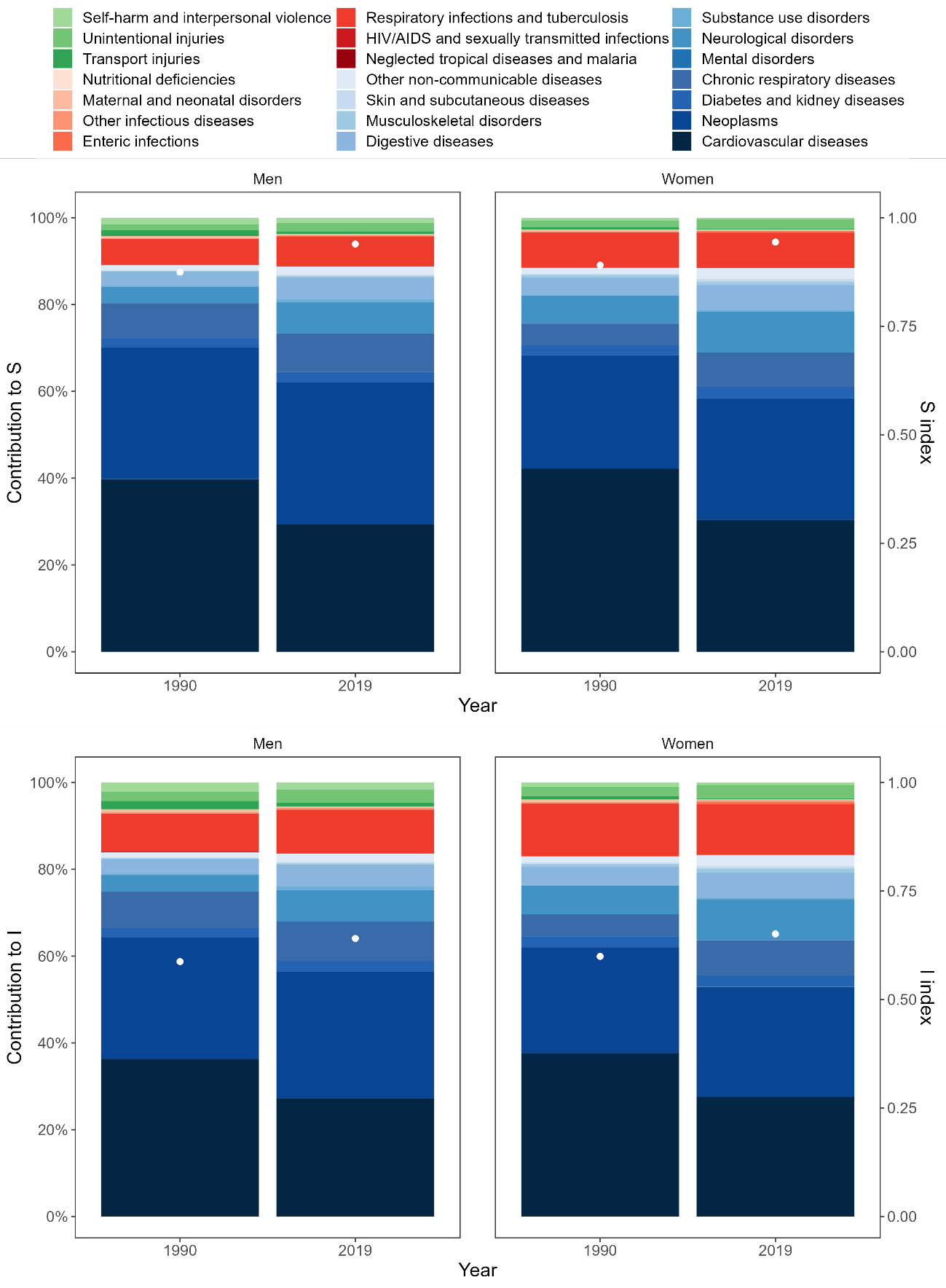
**

**Figure S13. Levels and cause-specific decompositions of the cause-of-death diversity (S) and inequality (I) indices in the United Kingdom for men and women (1990 and 2019).** Note: The scale of the cause-specific decomposition contributions is shown in the left-hand vertical axis, and the indices scale is in the right-hand one. In order to facilitate visualization, the 133 causes of death in level 3 have been aggregated in the graph into their corresponding level 2 categories. Source: Authors’ elaboration based on Global Burden of Disease/Institute for Health Metrics and Evaluation (GBD/IHME).

**
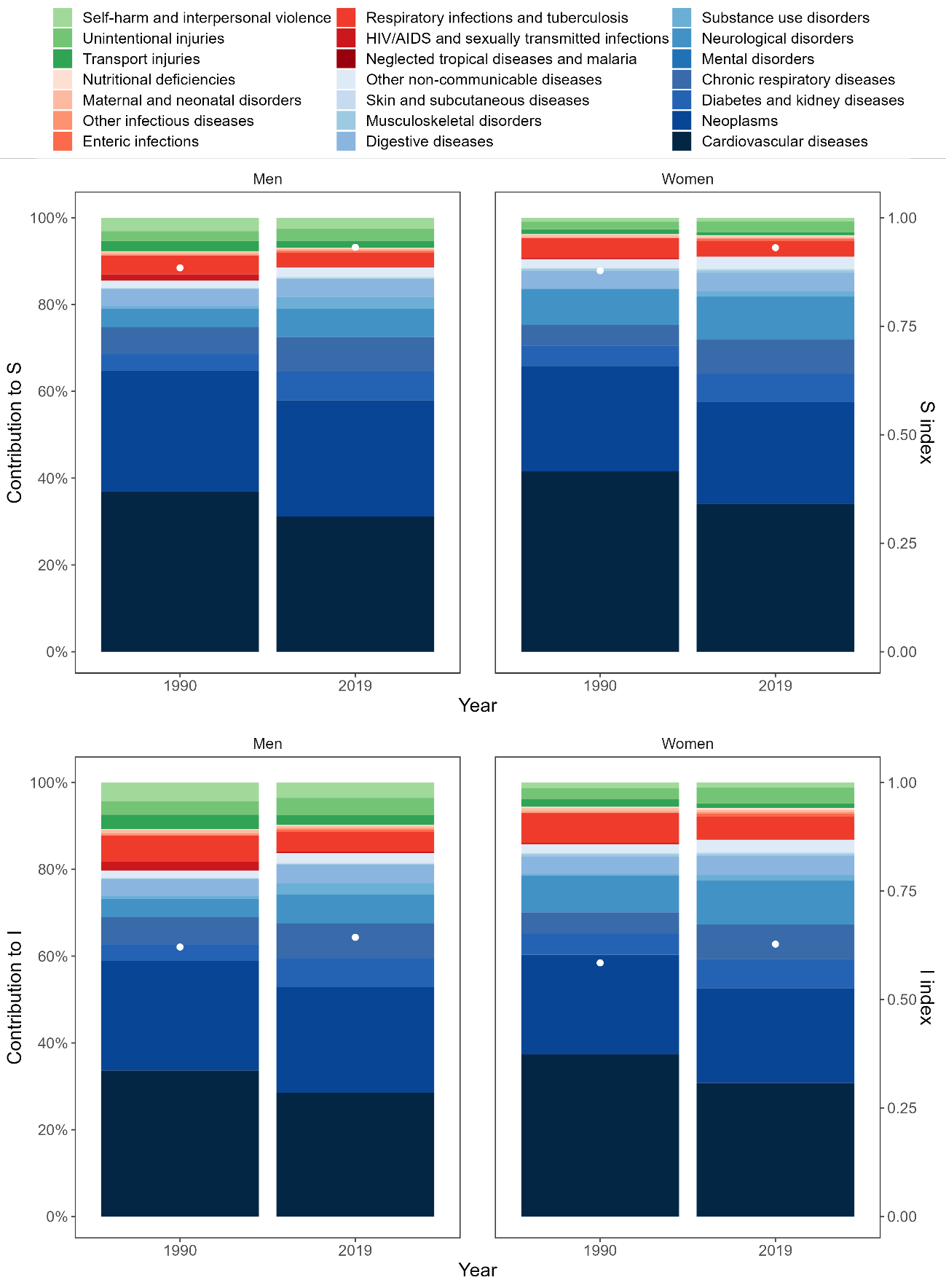
**

**Figure S14. Levels and cause-specific decompositions of the cause-of-death diversity (S) and inequality (I) indices in the United States of America for men and women (1990 and 2019).** Note: The scale of the cause-specific decomposition contributions is shown in the left-hand vertical axis, and the indices scale is in the right-hand one. In order to facilitate visualization, the 133 causes of death in level 3 have been aggregated in the graph into their corresponding level 2 categories. Source: Authors’ elaboration based on Global Burden of Disease/Institute for Health Metrics and Evaluation (GBD/IHME).

[[[Endnote #S1]]] Liu L, Villavicencio F, Yeung D, Perin J, Lopez G, Strong KL, et al. National, regional, and global causes of mortality in 5-19-year-olds from 2000 to 2019: a systematic analysis. Lancet Glob Health [Internet]. 2022;10(3):e337–47.
